# Human IL-22 receptor-targeted small protein antagonist suppress murine DSS-induced colitis

**DOI:** 10.1186/s12964-024-01846-w

**Published:** 2024-10-01

**Authors:** Milan Kuchař, Kristýna Sloupenská, Leona Rašková Kafková, Yaroslava Groza, Jozef Škarda, Petr Kosztyu, Marie Hlavničková, Joanna M. Mierzwicka, Radim Osička, Hana Petroková, Stephen I. Walimbwa, Shiv Bharadwaj, Jiří Černý, Milan Raška, Petr Malý

**Affiliations:** 1 Laboratory of Ligand Engineering, BIOCEV Research Center, Institute of Biotechnology of the Czech Academy of Sciences, Prumyslova 595, Vestec, 252 50 Czech Republic; 2 Department of Immunology, Faculty of Medicine and Dentistry, Palacky University Olomouc, Hnevotinska 3, Olomouc, 779 00 Czech Republic; 3 Department of Pathology, University Hospital Ostrava and Faculty of Medicine, University of Ostrava, Syllabova 19, Ostrava, 708 00 Czech Republic; 4 Laboratory of Molecular Biology of Bacterial Pathogens, Institute of Microbiology of the Czech Academy of Sciences, Videnska 1083, Prague, 14220 Czech Republic; 5 Laboratory of Structural Bioinformatics of Proteins, BIOCEV Research Center, Institute of Biotechnology of the Czech Academy of Sciences, Prumyslova 595, Vestec, 252 50 Czech Republic

**Keywords:** Interleukin-22, Inflammatory bowel disease, Experimental colitis, Immune suppression, Protein engineering

## Abstract

**Background:**

Human interleukin-22 (IL-22) is known as a “dual function” cytokine that acts as a master regulator to maintain homeostasis, structural integrity of the intestinal epithelial barrier, and shielding against bacterial pathogens. On the other hand, the overexpression of IL-22 is associated with hyper-proliferation and recruitment of pathologic effector cells, leading to tissue damage and chronic inflammation in specific diseases including inflammatory bowel disease (IBD). To study a role of IL-22-mediated signaling axis during intestinal inflammation, we generated a set of small protein blockers of IL-22R1 and verified their inhibitory potential on murine model of colitis.

**Methods:**

We used directed evolution of proteins to identify binders of human IL-22 receptor alpha (IL-22R1), designated as ABR ligands. This approach combines the assembly of a highly complex combinatorial protein library derived from small albumin-binding domain scaffold and selection of promising protein variants using ribosome display followed by large-scale ELISA screening. The binding affinity and specificity of ABR variants were analyzed on transfected HEK293T cells by flow cytometry and LigandTracer. Inhibitory function was further verified by competition ELISA, HEK-Blue IL-22 reporter cells, and murine dextran sulfate sodium (DSS)-induced colitis.

**Results:**

We demonstrate that ABR specifically recognizes transgenic IL-22R1 expressed on HEK293T cells and IL-22R1 on TNFα/IFNγ-activated HaCaT cells. Moreover, some ABR binders compete with the IL-22 cytokine and function as IL-22R1 antagonists in HEK-Blue IL22 reporter cells. In a murine model of DSS-induced acute intestinal inflammation, daily intraperitoneal administration of the best IL-22R1 antagonist, ABR167, suppressed the development of clinical and histological markers of colitis including prevention of mucosal inflammation and architecture deterioration. In addition, ABR167 reduces the DSS-induced increase in mRNA transcript levels of inflammatory cytokines such as IL-1β, IL-6, IL-10, and IL-17A.

**Conclusions:**

We developed small anti-human IL-22R1 blockers with antagonistic properties that ascertain a substantial role of IL-22-mediated signaling in the development of intestinal inflammation. The developed ABR blockers can be useful as a molecular clue for further IBD drug development.

**Supplementary Information:**

The online version contains supplementary material available at 10.1186/s12964-024-01846-w.

## Introduction

Interleukin-22 (IL-22), an innate lymphoid cell type 3 (ILC3) or Th17 signature cytokine, belongs to the interleukin-10 (IL-10) family [1] and it is mainly secreted by γδ T cells, innate lymphoid cells (ILC2 and ILC3), T helper (Th) 17 cells, and NKT cells [2]. IL-22 mediates its biological functions exclusively through a heterodimeric class II cytokine receptor comprising a high-affinity subunit, IL-22 receptor alpha (IL-22Rα or IL-22R1), and a low-affinity subunit, IL-10Rβ or IL-10R2 [3–5]. Notably, IL-22R1 expression is found exclusively on cell lineages originating from non-hematopoietic sources, such as the liver, kidney, pancreas, and barrier surfaces (i.e., lung, intestine, and skin) [1] whereas expression of IL-10R2 has been ubiquitously observed in various organs [2]. Available evidence establishes that IL-22 exhibits no affinity for IL-10R2, while a substantial binding affinity (K_D_ ranging from 1 ~ 20 nM) has been measured with the extracellular moiety of IL-22R1 [6, 7]. Also, attachment of IL-22 to IL-22R1 has been found to induce a conformational shift in IL-22 structure that promotes successive docking of the IL-10R2 subunit to the IL-22/IL-22R1 complex with a considerable recorded affinity (K_D_ = 7–45 µM) [8]. This structural information conveys that the initial attachment of IL-22 to IL-22R1 is required to initiate the secondary docking of the IL-10R2 subunit, thereby supporting the downstream signaling pathways in the target cells [9]. Importantly, the function of produced IL-22 is regulated by the soluble inhibitor IL-22 binding protein (IL-22BP, or IL-22R2), which is defined as a homolog of IL-22R1 and primarily released by myeloid cells [10, 11]. Likewise, the IL-22R1 expression is also maintained by concanavalin A or lipopolysaccharide (LPS) stimulation of hepatocytes, or IFNγ and TNFα in human skin cells [12], suggesting the IL-22 function may include dynamic variations linked to the expression of IL-22R1. Therefore, the IL-22/IL-22R1 system is involved in major crosstalk functions between epithelia and immune system, especially at the body barriers [1, 13].

In general, IL-22 has been characterized by its dual role − shielding or pathological functions during the initiation and progression of specific autoimmune and inflammatory diseases [14]. Under physiologic conditions, IL-22 can be beneficial by promoting epithelial repair or wound healing regeneration, and tissue integrity against pathogenic invaders [2, 15, 16]. Especially, IL-22 acts as a master regulator to maintain the homeostasis and structural integrity of the intestinal epithelial barrier [17, 18]. Also, in synergy with IL-17, IL-22 acts as a proinflammatory cytokine, which stimulates the upregulation of antimicrobial peptides in the epidermis including β-defensins and psoriasin (S100A7) [2, 19]. Furthermore, IL-22 is also demonstrated for protective functions in several diseases such as asthma, inflammatory bowel disease (IBD), and hepatitis [20–22]. Despite these beneficial functions, several studies suggest that IL-22 exhibits pathogenic functions depending on the discrete microenvironment and phase of the disease advancement [23–25]. In this context, the overexpression of IL-22 has been noted in several pathological conditions, as demonstrated by hyper-proliferation and recruitment of pathologic effector cells [1]. IL-22 overexpression is also reported to cause tissue damage and chronic inflammation in specific diseases including IBD [16].

Based on the published data, the IL-22/IL-22R1 system can be targeted by designed agonists or antagonists for the treatment of specific disorders or diseases. However, there is currently no Food and Drug Administration (FDA)–approved drug that directly targets IL-22 or IL-22R1, yet several drugs or formulations have been evaluated for selected diseases at different stages of development [13, 26]. Interestingly, most of the trial drugs in the process of being developed consist of modified recombinant IL-22 molecules with improved in vivo stability [27, 28]. For instance, Efmarodocokin alfa (UTTR1147A), an IL-22 used with crystallizable fragment (Fc) of human IgG4, showed effective engagement with IL-22R1 and demonstrated dose-dependent pharmacological activity in healthy volunteers under phase 1 trials [29]. In another clinical trial, Efmarodocokin alfa showed a long-term safety and tolerability profile in patients with moderate to severe ulcerative colitis [30]. Likewise, under a multicenter single-arm phase 2 study, designed F-652, a recombinant human IL-22, fused with an immunoglobulin constant region (IgG_2_-Fc) was noticed for favorable pharmacokinetics (PK) and pharmacodynamic (PD) properties, and was demonstrated as potential treatment for the acute Graft versus host disease (GVHD)-associated dysbiosis in the lower gastrointestinal (GI) tract in combination with systemic corticosteroids [28, 31]. In another clinical trial (phase 2a), fezakinumab (ILV-094) – an IL-22–neutralizing antibody, showed efficacy in patients with moderate to severe atopic dermatitis [32]. Moreover, probiotic *Lactobacillus*-expressing IL-22 has been suggested to deliver bioactive IL-22 directly to the intestinal mucosa to benefit patients with GVHD [33].

To interfere with cytokine-mediated cell signaling controlling the IL-23/IL-17 inflammatory axis, we have previously developed several collections of small binding proteins that function as IL-23 cytokine blockers [34], or IL-23 receptor (IL-23R) [35] and IL-17 receptor A (IL-17RA) antagonists [36, 37]. These small inhibitory ligands of 5 kDa were derived from the structure of the albumin-binding domain (ABD) of streptococcal protein G and identified by ribosome display selection from a highly complex combinatorial ABD library [38]. In this study, we report on the generation and characterization of ABD-derived protein blockers targeting IL-22R1 and discuss the role of IL-22 signaling inhibition during experimental murine model of intestinal inflammation induced by Dextran Sulfate Sodium (DSS).

## Materials and methods

### Cell lines

Human embryonic kidney cells (HEK293T), human keratinocytes (HaCaT), and HEK-Blue IL22 cells were cultured in high-glucose Dulbecco’s modified Eagle’s medium (DMEM) (BioSera, Cholet, France) supplemented by 10% fetal bovine serum (FBS) and streptomycin-penicillin solution (BioSera, Cholet, France), and incubated under 5% CO_2_ at 37 °C. Streptavidin-phycoerythrin (PE) was purchased from eBioscience, San Diego, CA, USA. Human anti-IL-22R1 APC-conjugated antibody (mouse IgG1 isotype) was procured from R&D Systems, Minneapolis, MN, USA, and mouse IgG1 APC-conjugated isotype control antibody MOPC-21 was obtained from Exbio Praha, a.s., Vestec, Czech Republic.

### **Assembly of ABD library**

A highly complex combinatorial NNK library (where N means any nucleotide, K is G or T only) derived from a scaffold of albumin-binding domain (ABD) was assembled by multiple PCR steps as previously reported and characterized [38]. Briefly, the ABD library was assembled using two oligonucleotides ABDLIB-setB1c and ABDLIB-setB2c (Table S1) and connected with cDNA coding for the tolA helical linker. Amplified by PCR was purified on 1% agarose (QIAquick Gel Extraction Kit, Qiagen, Hilden, Germany) and used for in vitro transcription and translation using PURExpress^®^ In Vitro Protein Synthesis Kit (NEB, Ipswich, MA, USA).

### Ribosome display

A modified ribosome display selection procedure was used for the assembled ABD library targeting recombinant human IL-22R1 receptor [34, 36]. For the preselection step, 3% BSA in TBS buffer (50 mM Tris, 150 mM NaCl, DEPC-treated water, pH 7.5), and for the selection steps, IL-22R1 (rhIL-22 Rα1, R&D Systems, Minneapolis, MN, USA) diluted in coating buffer (Bicarbonate/carbonate coating buffer, pH 9.6), were coated in wells of NUNC-immune MaxiSorp plates (Nunc A/S, Roskilde, Sjælland, Denmark). Then, all the wells were blocked using Protein-Free (PBS) Blocking Buffer (Thermo Fisher Scientific, Waltham, MA, USA) and incubated for 3 h at room temperature (RT). Following, the preselection wells were washed thrice and incubated with a mixture of in vitro translated ABD library (mRNA-ribosome-protein complex) and ABDwt (25 µg/ml) in WBT buffer (50 mM Tris-base, 150 mM NaCl, 50 mM MgAc, 0.05% Tween-20, DEPC-treated water, acetic acid to pH 7.5) at 8 °C for next 1 h under shaking conditions. Later, the unbound library from preselection wells was transferred into selection wells, washed thrice, and incubated for 1 h at 8 °C under shaking conditions. Finally, the selection wells were washed thrice with WBT buffer while mRNA-ribosome-protein complexes bound to the coated IL-22R1 protein were eluted using 200 µl of EB (50 mM Tris-base, 150 mM NaCl, 50 mM EDTA, Diethyl pyrocarbonate (DEPC)-treated water, pH by acetic acid to pH 7.5). Next, the total mRNA was extracted and purified using mRNA extraction kit. A total of three rounds of preselection/selection were performed with increasing stringency as follows: 1st round – plate coating with 25 µg/ml IL-22R1, washed 5 times by WBT buffer containing 0.05% Tween 20, 2nd round – 25 µg/ml IL-22R1, washed 10 times and 3rd round – 10 µg/ml IL-22R1, washed 10 times by WBT containing 0.1% Tween 20. Also, ABDWT protein was added as a competitor to the library in 1st and 3rd rounds only. Harvested mRNA was reversely transcribed by GoScript™ Reverse Transcriptase (Promega, Madison, WI, USA) and resulted in a DNA library that was finally inserted into the pET28b vector to form a plasmid library of selected DNA variants.

### Screening of IL-22R1-targeted ABD-derived variants

*Escherichia coli* BL21 (λDE3) BirA strain cells were transformed by the plasmid cDNA library. Individual bacterial colonies were picked up for overnight culturing in 2 ml LB broth at 37 °C with kanamycin (60 µg/ml) and chloramphenicol (30 µg/ml). Next day, 100 times diluted culture was further cultured, and protein production was induced with 1.5 mM isopropyl β-d-1-thiogalactopyranoside (IPTG) in the presence of 50 µM d-biotin (5 mM d-biotin solution in 10 mM Bicine buffer, pH 8.3) at 32 °C. After 4 h of incubation, the cells were harvested by centrifugation and obtained bacterial pellets were frozen. For the analysis, pellets were resuspended in PBS buffer and sonicated for 1 min using ultrasonic disruptor Misonix S3000 sonicator and centrifuged (18000×g) for 10 min at 4 °C. Cell supernatant was diluted 15,000 times in PBS/Tween-20/1%BSA (pH 7.4). For the identification of IL-22R1 specific binders, binding ELISA was used. Nunc MaxiSorp and PolySorp ELISA plates (Nunc A/S, Roskilde, Sjælland, Denmark) were coated with IL-22R1 (2.5 µg/ml). Then, MaxiSorp plates were blocked with Protein-Free (PBS) Blocking Buffer and PolySorp plates were blocked with 1% BSA in PBS-T (PBS, 0.05% Tween-20). Bacterial lysates containing in vivo biotinylated ABD protein variant were tested for binding to immobilized IL-22R1 using streptavidin-HRP conjugate (Pierce™ High Sensitivity Streptavidin-HRP, Thermo Fisher Scientific, Waltham, MA, USA). After 30 min incubation, TMB-Complete 2 (3,3’,5,5’-Tetramethylbenzidine, TestLine, Brno, Czech Republic) substrate was added and the reaction was stopped by 2 M sulfuric acid. The absorbance was measured at 450 nm using Epoch 2 microplate spectrophotometer (BioTek, Santa Clara, CA, USA).

### Competition ELISA

ABD-derived variants were purified from bacterial lysates in 50 mM Tris, 300 mM NaCl, and pH 8.0 buffer using affinity chromatography (Ni-NTA agarose, Qiagen, Hilden, Germany). For the competition assay, MaxiSorp plates were coated with human IL-22R1 (1 µg/ml), blocked with Protein-Free (PBS) Blocking Buffer, and purified ABD-derived variants were serially diluted in PBS-T with 1% BSA containing IL-22 cytokine (recombinant human IL-22 protein, Fc Chimera, Abcam, Cambridge, United Kingdom) at the concentration 1 nM, and cytokine binding was detected by mouse IgG1 anti-human IgG (Fc) HRP (1: 5 000, mouse monoclonal IgG to Fc part of human IgG heavy chain conjugated with horseradish peroxidase, Exbio Praha, a.s. Vestec, Czech Republic).

### Immunofluorescence staining of transfected HEK293T cells

Full-length human IL-22R1 cDNA coding for 574 amino acids protein containing a signal peptide (GenBank: BC029273.1) was cloned in pcDNA™6/myc-His A vector with added Kozak sequence. Before seeding the cells, 24-well plates (TPP, Trasadingen, Switzerland) were coated with 100 µl of 100 µg/ml poly-D-lysine (Gibco, Thermo Fisher Scientific, Waltham, MA, USA), incubated for 1 h at 37 °C, then washed 3 times with PBS and left to dry for 20 min at RT. Total 1.5 × 10^5^ cells were seeded 48 h or 2.5 × 10^5^ cells were seeded 24 h before the transfection. The complete growth medium was exchanged with DMEM without any supplements. Plasmid DNA and cationic polymer polyethylenimine (PEI) mixes were prepared in 50 µl of DMEM medium without supplements, mixed by inversion, incubated for 15 min at RT, and then applied dropwise into the wells. HEK293T cells were transiently transfected with PEI (PEI branched, MW 25,000; Sigma-Aldrich, St. Louis, MO, USA) in concentration 1 mg/ml, and in 4.5:1 PEI to plasmid DNA ratio, and 1 µg of DNA was used per each well. Transfected cells were incubated in 0.5 ml of DMEM medium without supplements for 18 h and then 0.5 ml of complete culture medium was added to each well. Two days after the transfection, particular protein variants at a concentration of 20 µg/ml were added to DMEM medium, and cells were incubated at 37 °C for 1 h and then 3 times washed with PBS. The primary staining mix contained Streptavidin–Alexa Fluor (AF) 568 conjugate (Thermo Fisher Scientific, Waltham, MA, USA) for detection of biotinylated ABR proteins and rabbit polyclonal anti-IL-22R1 antibody for the detection of IL-22R1. After 1 h incubation, cells were washed, and goat anti-rabbit IgG conjugated with AF488 (Abcam, Cambridge, United Kingdom) was added for next 1 h staining in the dark. Each well was washed 3 times with PBS and visualized using fluorescence microscopy with further image processing using ImageJ software.

### Competition staining with ABR proteins in the presence of IL-22 competitor

Staining procedure was performed in the same manner as described in section “Immunofluorescence staining of transfected HEK293T cells” with the following modifications: before the staining, cells were treated with IL22 (Abcam, Cambridge, United Kingdom) for 1 h (cells were kept in cell incubator at 37 °C) which was followed by addition of ABR proteins into wells with previously treated and non-treated cells. Cells were again incubated for 1 h in cell incubator at 37 °C. After the incubation, cells were subjected to staining and visualized under microscope.

### Binding kinetics measured with LigandTracer

HEK293T cells were transiently transfected with 1 mg/ml PEI (MW 25,000) and the PEI – DNA ratio was 4.5:1. Prior to 24 h before the transfection, 3 ml of cell suspensions (1 × 10^6^ cells) was seeded on 100 mm cell dishes (Nunclon^TM^, Sigma-Aldrich, St. Louis, MO, USA) in the designated area (marked as target area) and incubated overnight in a tilted position. Before seeding the cells, dishes were coated with 2.3 ml of 100 µg/ml poly-D-lysine, incubated for 1 h at 37 °C, then washed 3 times with PBS and left to dry for 20 min. The next day, the medium was exchanged into a medium without supplements and cells were transfected. Plasmid DNA and PEI mixes were prepared in 300 µl of DMEM medium without supplements, mixed by inversion and incubated for 15 min at RT. Transfected cells were incubated in 3 ml of DMEM medium without supplements in a tilted position for 18 h and then 6 ml of complete growth medium was added. Cells were incubated at the horizontal position for the next 24 h. For the measurement of ABR proteins kinetics, the LigandTracer Green Line (Ridgeview Instruments AB, Uppsala, Sweden) with Red - NIR (632–671 nm) detector was used. The fluorescence signal was detected using Streptavidin-APC conjugate (Thermo Fisher Scientific, Waltham, MA, USA). The evaluation of binding kinetics was done using TraceDrawer 1.7.1 software. Kinetic parameters (ka, kd, KD) were calculated using ‘One-to-one’ or ‘One-to-one depletion corrected’ evaluation methods.

### Binding of ABR proteins to HaCaT cells

HaCaT cells were cultured in DMEM with 2 mM L-glutamine, 5.4 g/l glucose, 10% heat-inactivated fetal bovine serum, 100 U/ml Penicillin, 100 µg/ml Streptomycin at 37 °C in 5% CO_2_. After overnight incubation, 10 ng/ml TNFα (Abcam, Cambridge, United Kingdom) and 10 ng/ml IFNγ (Abcam, Cambridge, United Kingdom) were added for 24 h according to the previous study [39]. Biotinylated ABR variants were added in DMEM at a concentration 20 µg/ml (≈ 500 nM) for 1 h at 37 °C. Cells were washed 3 times with PBS. Rabbit anti-IL-22 polyclonal Ab (Abcam, Cambridge, United Kingdom) and rabbit anti-SARS-CoV-2 S1 RBD polyclonal antibody (RayBiotech, Peachtree Corners, GA, USA) as an isotype control were diluted 100 times in PBS with 1.5% BSA and added to cells and incubated for 1 h at RT. After 3 times washing with PBS, Streptavidin (AF568) (Invitrogen, Waltham, MA, USA) or goat anti-rabbit IgG H&L (AF488) pAb (Abcam, Cambridge, United Kingdom) were added to cells and incubated for 1 h at RT. Both reagents were diluted 200 times in PBS with 1.5% BSA. Finally, HaCaT cells were washed 5 times with PBS. Then, cell imaging was made with an Olympus CKX41 Inverted Phase Contrast Microscope (Olympus, Tokio, Japan). For the estimation of binding kinetics by LigandTracer, Streptavidin-APC conjugate was used for ABR proteins detection.

### Flow cytometry

Streptavidin-phycoerythrin (PE) was obtained from eBioscience, San Diego, CA, USA. Human anti-IL-22R1 APC-conjugated antibody (mouse IgG1 isotype) was purchased from R&D Systems, Minneapolis, MN, USA and mouse IgG1 APC-conjugated isotype control antibody MOPC-21 was obtained from Exbio Praha s.r.o., Vestec, Czech Republic. Cultured HEK-Blue IL22 and HEK-293T cells were collected and washed in HEPES-buffered salt solution (HBSS buffer; 10 mM HEPES (pH 7.4), 140 mM NaCl, 5 mM KCl) supplemented with 2 mM CaCl_2_, 2 mM MgCl_2_, 1% (w/v) glucose, and 1% (v/v) FCS (cHBSS buffer). 2 × 10^5^ cells/sample in cHBSS buffer were incubated with 1 µg/ml of biotin-labeled ligands (ABRs and ABD) for 30 min at 4 °C. After washing with cHBSS buffer, cells were incubated with PE-labeled streptavidin (diluted 1:400) for 30 min at 4 °C. Cells were washed, resuspended in cHBSS buffer, and analyzed by flow cytometry using a FACS LSR II instrument (BD Biosciences, San Jose, CA, USA) in the presence of 1 µg/ml of Hoechst 33258. Data was processed using the FlowJo software (BD Biosciences, Ashland, OR, USA) and appropriate gating was used to exclude debris, cell aggregates, and dead cells (Hoechst 33258-positive staining). Binding data are expressed as mean fluorescence intensity (MFI) values. For antibody binding, 2 × 10^5^ cells/sample were incubated in cHBSS buffer with anti-IL-22R1 APC-conjugated antibody (diluted 1:100, final concentration 0.1 µg/ml) or IgG1 isotype control (diluted 1:100) for 30 min at 4 °C. After washing, cells were resuspended in cHBSS buffer, and analyzed by flow cytometry as described above.

### HEK-Blue IL22 reporter cell inhibition assay

HEK-Blue IL22 Reporter Cell line (InvivoGen, San Diego, CA, USA) was cultured in DMEM with 2 mM L-glutamine, 5.4 g/l glucose, 10% heat-inactivated fetal bovine serum, 100 U/ml Penicillin, 100 µg/ml Streptomycin, 100 µg/ml Normocin, 10 µg/ml Blasticidin, 10 µg/ml Puromycin, 100 µg/ml Zeocin at 37 °C in 5% CO_2_. For the inhibition assay, HEK-Blue IL22 cells were seeded to a NUNC sterile 96-well plate (Nunc A/S, Roskilde, Sjælland, Denmark), 36,000 cells per well in a volume of 180 µl. HEK-Blue IL22 cells were grown on a surface treated with Poly-D-Lysine (Gibco, Thermo Fisher Scientific, Waltham, MA, USA) to ensure uniform attachment without cell clamping which was observed on an untreated surface. Inhibition assay was carried out in DMEM with 2 mM L-glutamine, 5.4 g/l glucose, 100 U/ml Penicillin, 100 µg/ml Streptomycin, 100 µg/ml at 37 °C in 5% CO_2_. Cells were incubated with human IL-22 (Abcam, Cambridge, United Kingdom) for 22 h in the presence of different concentrations of ABR variants. Neutralizing anti-IL-22 monoclonal antibody IL22JOP was used as a positive control, while ABDWT protein and irrelevant rat IgG2aκ anti-IL-6 monoclonal antibody (BioLegend, San Diego, CA, USA) were used as negative controls. ABR variants were serially diluted in sterile PBS by 5 times per step with the highest concentration being 120 nM and added to cells in 20 µl volume. After 22 h, 20 µl of cell supernatant was mixed with 180 µl of the Quanti-BlueTM Solution (InvivoGen, San Diego, CA, USA) to detect secreted alkaline phosphatase (SEAP) that was produced by HEK-Blue IL22 cells in response to human IL-22 stimulation. Cell supernatant was incubated with Quanti-Blue Solution for 1 h at 37 °C in the dark. Absorbance was measured at 620 nm with Epoch 2 microplate spectrophotometer. HEK-Blue IL22 reporter cells were incubated with 3 ng/ml (≈ 0.2 nM) of human IL-22 and varied concentration of ABR variants. Secreted embryonic alkaline phosphatase (SEAP) level for each ABR concentration, as detected by measuring absorbance at 620 nm, was compared to the SEAP level while human IL-22 was added in the absence of any other protein.

### Molecular modeling

We modeled the structure of the ABD-derived variants using the MODELLER 9v14 software suite [40] using the structure of the wild type ABD (pdb id 1gjt [41]) as the template. The IL-22R1 structure was obtained from the crystal structure of the IL-22/IL-22R1 complex (pdb id 3dlq [9]). For protein-protein docking with flexible side chains, we utilized a local version of the ClusPro server [42, 43], using chain B from the 3dlq structure (residues missing in the template were modeled using the automodel function of MODELLER taking the best scoring model corresponding to IL-22R1 domains 1 and 2, residues 18 to 228, according to the UniProt [44] record Q8N6P7) as the receptor and the modeled ABR variants as ligands. The docking results were visualized with PyMOL version 2.6.0 (The PyMOL Molecular Graphics System, Schrödinger, LLC, New York, NY, USA).

### Animals and experimental design

8–9 weeks old female C57BL/6 mice (AnLab, Prague, Czech Republic) weighing between 18 and 22 g (weight before treatment) were kept under standardized conditions at temperature between 21 and 22 °C, a 12:12-h light/dark cycle, ad libitum access to food and water. Mice were permitted to acclimatize for 1 week before starting the experiments. Mice were randomly divided into experimental groups and marked with ear tags.

### Induction of acute colitis

Acute colitis was induced by giving drinking water containing 2.5% (w/v) DSS (MW approximately 40 kDa; TdB Labs, Uppsala, Sweden) for 4 days. At the end of the experiment, the animals were anesthetized, bled out, and euthanized by cervical dislocation. The length of the colon was measured between the caecum and proximal rectum. Serum was isolated from blood. Colon was dissected into pieces for quantitative real-time RT-PCR (qRT-PCR) and histochemistry. All experiment protocols were approved by Ethics Committee of the Faculty of Medicine and Dentistry (Palacky University Olomouc, Czech Republic), and the Ministry of Education, Youth and Sports, Czech Republic (MSMT-10947/2021-3).

### Preventive treatment of mice by intraperitoneal application (i.p.) of binders

C57BL/6 mice were divided into three groups. Group 1 (naive) – consisting of 10 naive untreated mice. Group 2 (DSS + ABR167) – consisting of 19 mice treated by i.p. administration of 25 µg of ABR167 per mouse every 24 h starting 3 days before DSS-colitis induction and continuing for another 4 days together with DSS administration, and the Group 3 (DSS induction control) – consisting of 13 mice exposed to DSS without the therapy.

### RNA isolation, reverse transcription, and qRT-PCR

Dissected colon tissues were stored in RNA later (Invitrogen, Waltham, MA, USA) at -80 °C. Pre-weighed colons were homogenized using Qiashredder (Qiagen, Hilden, Germany) and total RNA was extracted using RNeasy Plus Mini Kit (Qiagen, Hilden, Germany). Column DNase treatment (gDNA Eliminator spin column, Qiagen, Hilden, Germany) was used to eliminate potential DNA contamination. RNA purification was performed via lithium chloride method [45] to remove the traces of DSS. Extracted RNA was reversely transcribed into cDNA using the gb Elite Reverse Transcription Kit (Generi Biotech, Hradec Kralove, Czech Republic). qRT-PCR was performed in triplicates using gb SG PCR Master Mix (Generi Biotech, Hradec Kralove, Czech Republic) on LightCycler 480 System (Roche, Basel, Switzerland). All primers (6 µM) used in this study are listed in Table S2. Ct values were normalized to the reference gene, GAPDH, and the relative RNA expression was calculated by 2^−ΔΔCt^ method [46].

### Measurement of serum cytokine concentration

Concentration of cytokines IL-1β, IL-4, IL-6, IL-12p40, IL-18, IL-22, IL-23 and TNFα in serum was measured using fluorescent microbeads (LEGENDplex™ Custom Human Assay, Biolegend, San Diego, CA, USA) conjugated with antibody targeting particular cytokine according to manufacturer´s instructions. Briefly, serum and mixture of cytokine standards were applied into 96-well plate and diluted 1:1 in assay buffer. Mixture of conjugated beads was added into wells and incubated for 2 h at room temperature. Wells were washed and mixture of biotinylated detection antibodies targeting all selected cytokines was added. Plate was incubated for 90 min at RT. Then, streptavidin conjugated with phycoerythrin was added and plate was incubated for 30 min at RT. After washing, beads were analyzed using flow cytometer SONY SP6800 (Sony, Tokio, Japan).

### Histochemistry

Formalin-fixed, paraffin embedded tissues were cut, stained with hematoxylin and eosin (H&E) (Merck, Darmstadt, Germany), classified by a professional pathologist, and verified by medical doctor without prior knowledge of clinical parameters using BX43 microscope equipped with CCD camera (Olympus, Tokio, Japan). The representative areas of the most intense mucosal alterations were selected in each section. These areas were scored for given parameters as summarized in the Table S5.

### Statistical analysis

All statistical analyses were performed using GraphPad Prism 8 Software (GraphPad Software Inc., San Diego, CA, USA) or OriginLab version 2023b (OriginLab Corporation, Northampton, MA, USA). All data sets were verified for normal (Gaussian) distribution by Normality test. We performed Kruskal-Wallis one-way ANOVA followed by Dunn´s multiple comparisons test; *P < 0.05, **P < 0.01, ***P < 0.001.

## Results

### Directed evolution of ABD variants targeting human IL-22R1

**Fig. 1:**
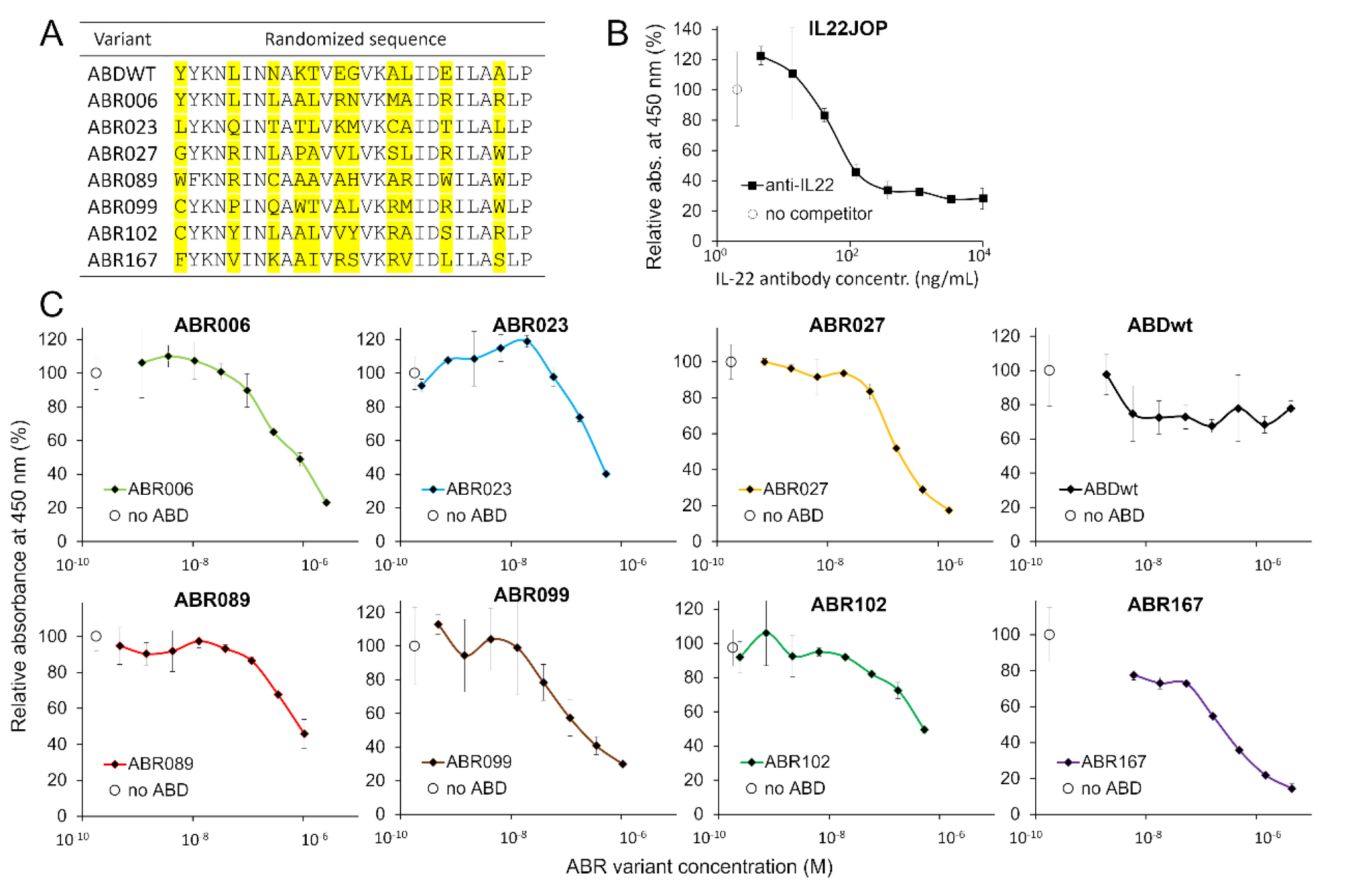
**(A)** Amino acid sequences of the identified variants as well as ABDwt are shown in the table with marked randomized positions (yellow). **(B)** Binding of IL-22 to IL-22R1 in the presence of neutralizing antibody tested by competition ELISA. MaxiSorp plate was coated with IL-22R1 (IgG Fc chimera) at concentration 2.5 µg/mL in carbonate buffer (pH 9.6). Serially diluted anti-IL-22 monoclonal antibody (clone IL22JOP) prepared in PBS (pH 7.4, 0.05% Tween, 1% BSA) with diluted IL-22 (His-tag) at constant concentration 1nM. IL-22 binding was detected with anti-His-tag monoclonal antibody conjugated with HRP. All samples were measured in triplicates, error bars represent the standard deviation. **(C)** ELISA-based competition assay was performed with purified protein variants. IL-22R1 was immobilized on MaxiSorp plate, serially diluted ABR variants and negative control ABDwt competed with IL-22 at constant concentration (1 nM) for binding to the receptor and the resulted curves are compared to the average value corresponding to cytokine binding in the absence of the ABR competitor (no ABR). IL-22-Fc binding was detected by mouse Anti-Hu IgG (Fc) HRP and average values with standard deviation error bars are presented

To generate ABD-derived variants that inhibit signaling function of human IL-22R1, we used directed evolution and employed MaxiSorp immune-plate for ribosome display selection as ELISA experiment demonstrated the accessibility of IL-22 to the cytokine binding site on IL-22R1 once coated on 96-well plate (Fig. S1). Three-round ribosome display resulted in the generation of ABD library containing cDNA of the selected variants. This library was cloned into pET28b plasmid and used to transform in *E .coli* cells. ELISA screening of bacterial lysates resulted in the selection of ABD variants with preferential binding to the IL-22R1 called ABR proteins, which were purified and re-tested in equal concentration using PolySorp plate (Fig. S2). Following this approach, a total of 170 ABR variants were assessed for the specific binding to commercial IL-22R1, and 39 variants with considerable binding affinity were further verified by sequence analysis (Fig. 1A). This identified 24 unique variants expressing a full-length protein, and 16 candidate variants were selected for further characterization whether they compete with IL-22 cytokine.

### Engineered ABR variants block IL-22 binding to immobilized and cell-surface expressed IL-22R1

To evaluate the ability of the engineered ABR variants to inhibit binding of IL-22 to IL-22R1, we performed the competition ELISA assay. The blocking effect of the selected candidates was monitored on the immobilized IL-22R1 receptor on the MaxiSorp plate. To validate the assay, neutralizing anti-IL-22 monoclonal antibody IL22JOP was used to block binding of IL-22 to the coated recombinant IL-22R1 (Fig. 1B). The collected results supported 12 ABR variants with considerable competition effect using ELISA and 7 of them were selected for further characterization (Fig. 1C).

**Fig. 2:**
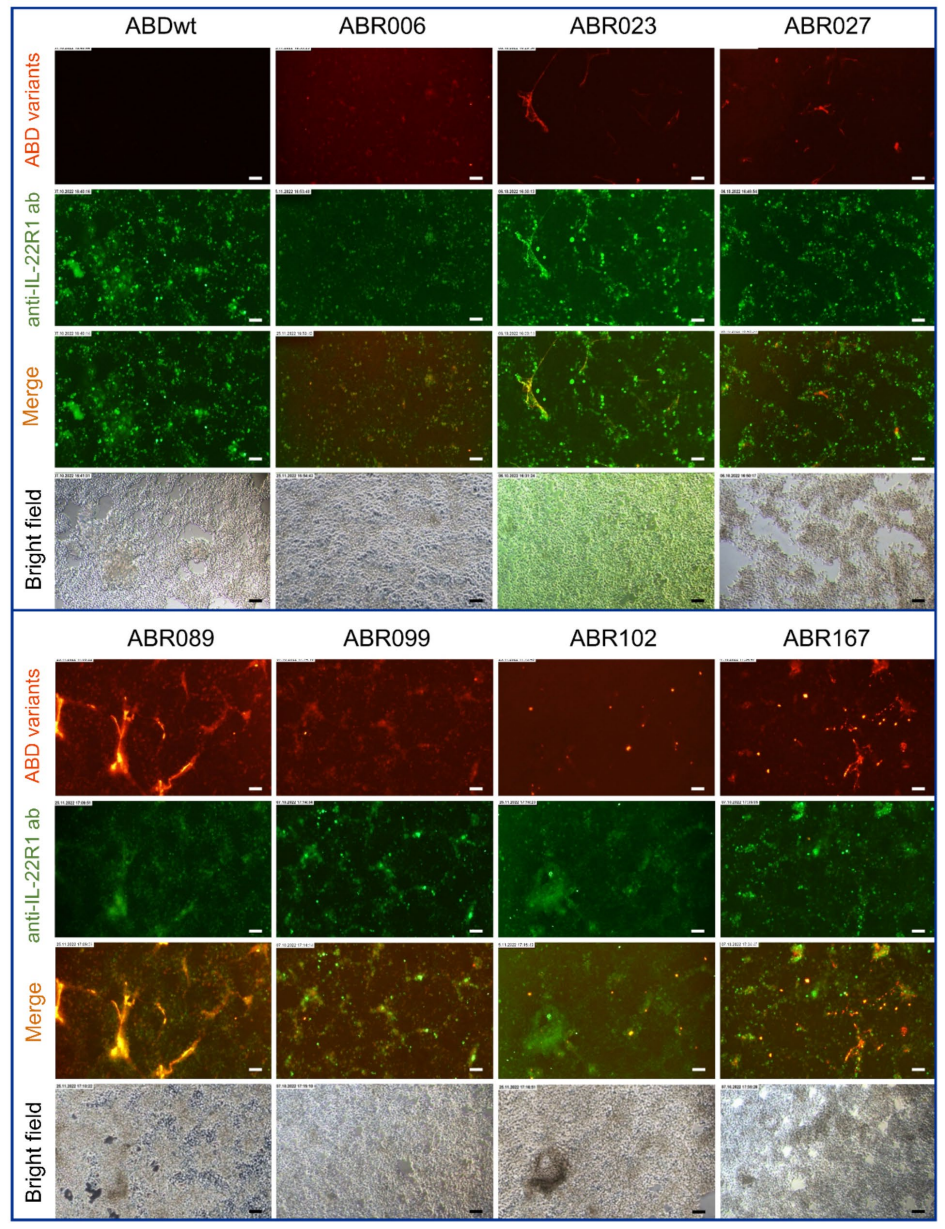
Detection of IL-22R1 expression on transfected HEK293T cells and binding of ABR variants to IL-22R1. Representative images of HEK293T cells expressing human IL-22R1 on the surface. Cells were incubated with biotinylated ABR variants (ABR006, ABR023, ABR027, ABR089, ABR099, ABR102, and ABR167) and stained with Streptavidin-AF568 (STV-AF568 conjugate) and anti-IL-22R1 antibody (anti-IL-22R1-AF488)

Next, we used fluorescent microscopy to test the ability of ABR variants to recognize IL-22R1 on the surface of transfected HEK293T cells. To test IL-22R1 expression on HEK293T transfected cells, rabbit polyclonal anti-IL-22R1 antibody was used and its specificity verified on HEK293T transfected, mock-transfected and non-transfected cells (Fig. S3). Seven ABR variants (ABR006, ABR023, ABR027, ABR089, ABR099, ABR102, and ABR167) recognized IL-22R1 expressed on the surface of HEK293T cells (Fig. 2) in contrast to the negative staining on mock-transfected cells (Fig. S4).

**Fig. 3:**
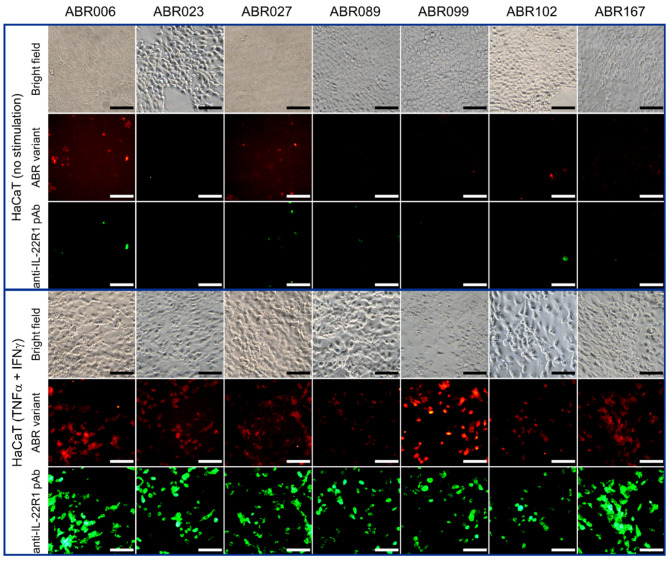
Fluorescence microscopy images demonstrating ABR variant’s binding to the IL-22R1 on stimulated HaCaT cells. HaCaT cells were stimulated with TNFα and IFNγ for 24 h. Double-staining of ABR proteins detected by Streptavidine-AF568 conjugate (red) and rabbit anti-IL-22R1 pAb-AF488 (green) was carried out

To further confirm the specificity of the ABR proteins to recognize IL-22R1, HaCaT cells expressing endogenously IL-22R1 upon double-stimulation with TNFα and IFNγ were used for immunofluorescence detection. Control experiments demonstrating the specificity of the used rabbit polyclonal anti-IL-22R1 antibody on stimulated/unstimulated HaCaT cells were performed (Fig. S5, Fig. S6). The immunofluorescence staining performed for 7 ABR variants positively staining HEK293T transfected cells also exhibited specific binding to IL-22R1-expressing HaCaT cells upon the double-stimulation with TNFα and IFNγ (Fig. 3), while demonstrating no positivity for the unstimulated HaCaT cells.

### Generation of ABR089 and ABR099 variants with cysteine-to-serine substitution

For the selection of protein variants targeting human IL-22R1, ABD-derived combinatorial library with more restricted degenerate codon (NNK type) was used, thus covering 32 codons. This approach is based on the randomization of single nucleotides (N means any nucleotide, K is G or T only) and results in the generation of codons for all 20 amino acid residues, including Cysteine residues and one of stop codons. As shown in Fig. 1A, four of seven identified inhibitory variants (ABR023, ABR089, ABR099, and ABR102) contain a Cysteine residue in the randomized positions. As ABR023 and ABR102 did not demonstrate a substantial binding to HEK293T transfected cells (Fig. 2) and stimulated HaCaT cells (Fig. 3), we used sequences of ABR089 and ABR099 cDNA for the substitution of Cysteine for Serine residue. This was motivated to prevent from an occasional dimer formation that can, in some cases, block the accessibility to the binding site, thus suppressing the inhibitory function of these proteins. We used site directed mutagenesis to construct ABR089S protein with C27S mutation and ABR99S containing C20S substitution. The analysis of data from ELISA demonstrated that ABR089S as well as ABR099S retained the binding to IL-22R1 receptor in comparison to the non-mutated ABR089 and ABR099 variants (Fig. S7).

### Inhibition of IL-22-mediated signaling by ABR in HEK-Blue IL22 reporter cells

**Fig. 4:**
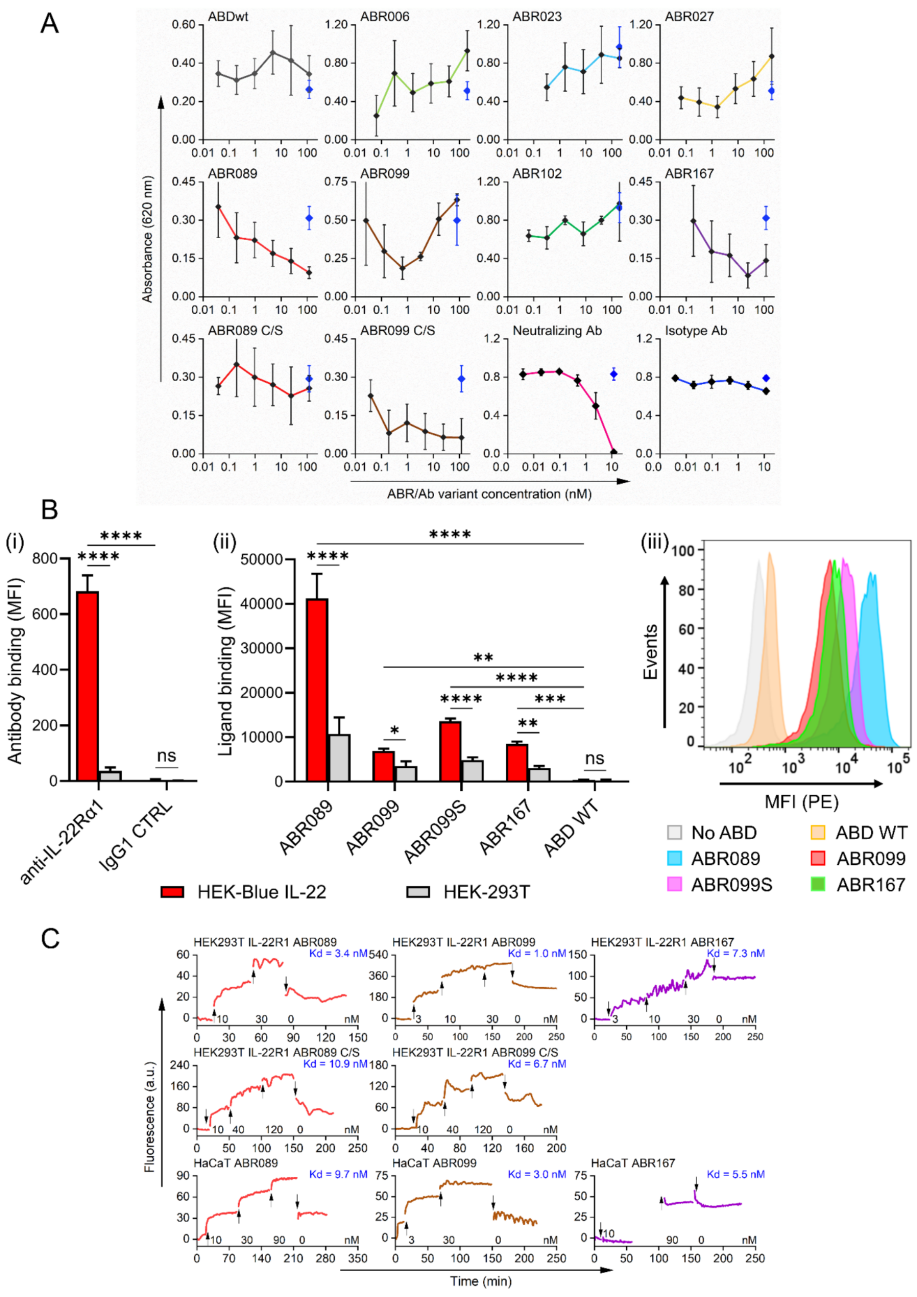
**(A)** Inhibitory function of ABR proteins tested on HEK-Blue IL22 reporter cells. SEAP reporter secretion was induced by 3 ng/ml hIL-22 and measured at 620 nm. Blue rhombs and black circles joined by a dotted line on the graph represent the absorbance in the absence and presence of varying ABR or antibody concentrations, respectively. Background absorbance in the absence of both human IL-22 and ABR variants was subtracted from all values depicted on the graphs. **(B)** ABR ligands bind to HEK cells expressing the human IL-22 receptor. (i) HEK-Blue IL-22 cells stably expressing the human IL-22 receptor (IL-22R) and HEK-293T control cells not expressing IL-22R were incubated with an APC-conjugated anti-IL-22R1 antibody or APC-conjugated IgG1 isotype control antibody (IgG1 CTRL) and analyzed by flow cytometry. The amounts of bound antibodies are expressed as MFI values. (ii) HEK-Blue IL-22 and HEK-293T control cells were incubated with 1 µg/ml of selected biotinylated ABR variants or with biotinylated ABD WT, which served as a negative control, for 30 min at 4 °C. Cell-bound proteins were analyzed by flow cytometry and the binding data are shown as MFI values (**C**). (i, ii) Each bar represents the mean value with SD of two independent experiments performed in duplicate (ns, *p* > 0.05; *, *p* < 0.05; **, *p* < 0.01; ***, *p* < 0.001; ****, *p* < 0.0001; ANOVA). (iii) Binding of ABR ligands to HEK-Blue IL-22 cells. ABD WT was processed in parallel and used as a negative control. A typical flow cytometry histogram from a representative binding experiment is shown. **(C)** Binding of ABR proteins to IL-22R1-transfected HEK293T cells and HaCaT cells detected using LigandTracer Green. Representative binding curves from LigandTracer measurements are shown for variants ABR089, ABR099, and ABR167. For ABR089 and ABR099, binding of the proteins to the IL-22R1 expressed on transfected HEK293T cells was also measured for C/S proteins

To verify a possible antagonistic effect of ABR ligands, we used HEK-Blue IL22 reporter cells which are available for monitoring of IL-22-mediated signaling. Human IL-22 triggers IL-22R1 assembly with subsequent STAT3 phosphorylation in HEK-Blue IL22 cells, followed by secretion of SEAP into cell supernatant. Seven ABR proteins (ABR006, ABD023, ABD027, ABR089, ABR099, ABR102, and ABD167) were tested for their ability to inhibit human IL-22 signaling in HEK-Blue IL22 reporter cells. Selected ABR variants that were tested in the cell inhibitory assay, outcompeted human IL-22 binding to IL-22R1 in competitive ELISA (Fig. 4A). Anti-IL-22 neutralizing monoclonal antibody IL22JOP was used as a test-validating positive control, while ABDwt and rat IgG2aκ isotype antibody as negative controls. Two ABR variants, ABR089 and ABR167, showed a persuasive inhibitory trend (Fig. 4A). The decrease in the absorbance up to 70% was observed for the concentration range 0.2–129 nM for ABR089 and 0.2–24 nM for ABR167 in comparison to the signal of human IL-22 alone. On the contrary, ABDwt did not demonstrate concentration dependent decrease in absorbance, indicating that inhibitory trends observed for ABR089 and ABR167 were not biased by ABD scaffold properties or cell cytotoxicity. Thus, ABR089 and ABR167 demonstrated the ability to inhibit human IL-22 down-stream signaling in HEK-Blue IL22 reporter cells which is in correlation with the inhibitory effect demonstrated by competition ELISA (Fig. 1C). As shown in Fig. 4A, the substitution of Cysteine to Serine residue in ABR99S protein substantially improved the blocking efficacy of this variant, while Cysteine-to-Serine substitution in ABR89S led to the loss of the inhibitory function. To further demonstrate the statistical significance of the observed inhibitory trend for ABR89, ABR99S and ABR167 proteins, we provide p values for 3 highest concentrations in comparison to Blue rhomb control values in Fig. 4A: ABR089 (*p* < 0.0001, < 0.0002, < 0.0010); ABR099S (*p* < 0.0001; <0.0001; <0.0001); ABR167 (*p* < 0.0006; <0.0001; <0.0036); and values for neutralizing antibody IL22JOP (*p* < 0.0001; <0.0016; <0.1945) analyzed by one-way ANOVA (*p* < 0.05).

### Binding of ABR proteins to HEK-Blue IL22 cells tested by flow cytometry

HEK-Blue IL22 cells, reported as a sensitive cell line to promote IL-22-mediated signaling, were used to verify the specificity of selected ABR protein candidates by flow cytometry. As shown in Fig. 4B(i), HEK-Blue IL22 cells substantially express IL-22R1 as documented by the binding of anti-human IL-22R1 antibody in contrast to isotype IgG1 antibody control or non-transfected HEK 293T cells. As further shown in Fig. 4B(ii), ABR variants bind to HEK-Blue IL22 cells with the strongest binding of ABR089 protein. The non-randomized ABD parental protein used as a negative control did not bind to these cells, thus documenting the specificity of the tested ABR binders (Fig. 4B(ii-iii)).

### Binding kinetics and affinity measurements

Initially, we tested binding of anti-IL-22R1 antibody to HEK-Blue IL22, HEK293T and HaCaT cells (Fig. S8, Table S3). The obtained results confirmed the presence of the IL-22R1 on the cell surface in all three cell types, and hence, all three cell lines can be used to measure kinetics of ABR ligands. Based on this observation, we selected HEK293T cells transfected with IL-22R1-pcDNA6 and HaCaT cells for the binding kinetics and affinity measurement for the selected ABR variants under similar conditions (Fig. 4C). Kinetic parameters were measured on HEK293T and HaCaT cells for all ABR variants presented in Table S4. The collected results showed that ABR089, ABR099, and ABR167 have Kd = 3.4 nM, Kd = 1.0 nM, and Kd = 7.3 nM as well as Kd = 9.7 nM, Kd = 3.0 nM, and Kd = 5.5 nM for HEK293T and HaCaT cells, respectively. In addition, C/S substitution on the ABR089 and ABR099 did not seem to significantly affect the binding capacity to HEK293T cells (Fig. 4C).

### Staining of IL22R1-expressing HEK293T cells with ABR proteins in the presence of IL-22 competitor

**Fig. 5:**
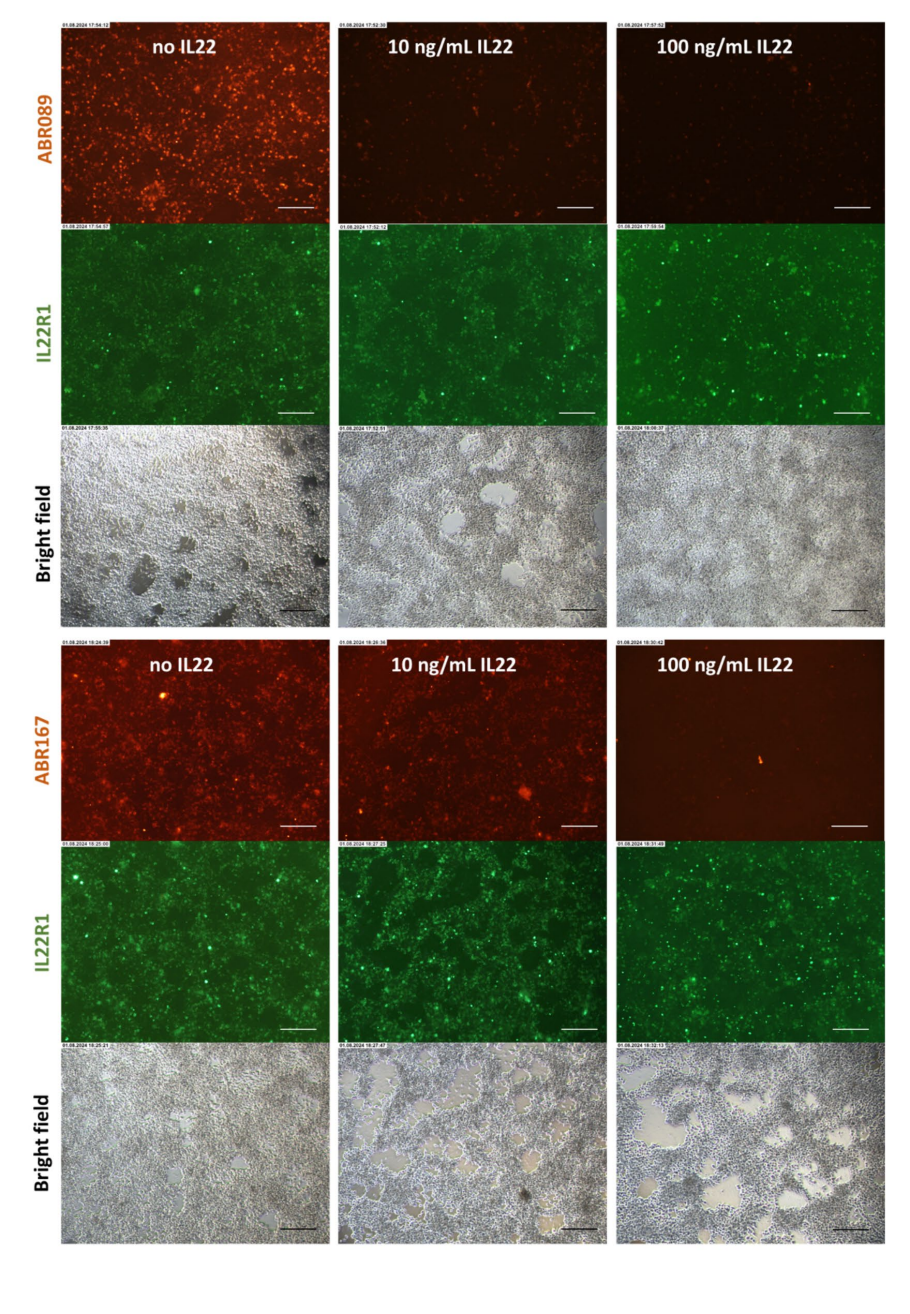
Competition staining of IL22R1 with ABR proteins in the presence of IL-22 competitor. IL22R1-transfected HEK293T cells were treated with increasing concentrations of IL-22 (10 ng/mL and 100 ng/mL) or were not treated (no IL22). Treatment of cells with IL-22 was done for 1 h followed by 1 h incubation with ABR089 or ABR167 into wells with treated and non-treated cells. Binding of biotinylated ABR proteins was performed using Streptavidin–AlexaFluor 568 conjugate. Magnification is 10x

To further demonstrate the specificity of ABR blocking variants for cell-expressed IL-22R1, we used a competition format in which ABR089 and ABR167 biotinylated proteins competed with IL-22 cytokine for binding to IL-22R1 expressed on HEK293T cell transfectants. As shown in Fig. 5, IL-22 in concentration 10 ng/ml as well as 100 ng/ml substantially decreased the binding of ABR089 protein to IL-22R1-transfected HEK293T cells in contrast to staining without the competitor. In the case of ABR167 protein, significant reduction of staining efficiency was observed at the concentration 100 ng/ml of the competing IL-22 cytokine. This clearly demonstrate that both these ABR variants share an overlapping binding site on the IL-22R1 with the cognate cytokine ligand, thus confirming the IL-22R1 specificity of the used protein binders.

### Prediction of binding modes by molecular modeling

**Fig. 6:**
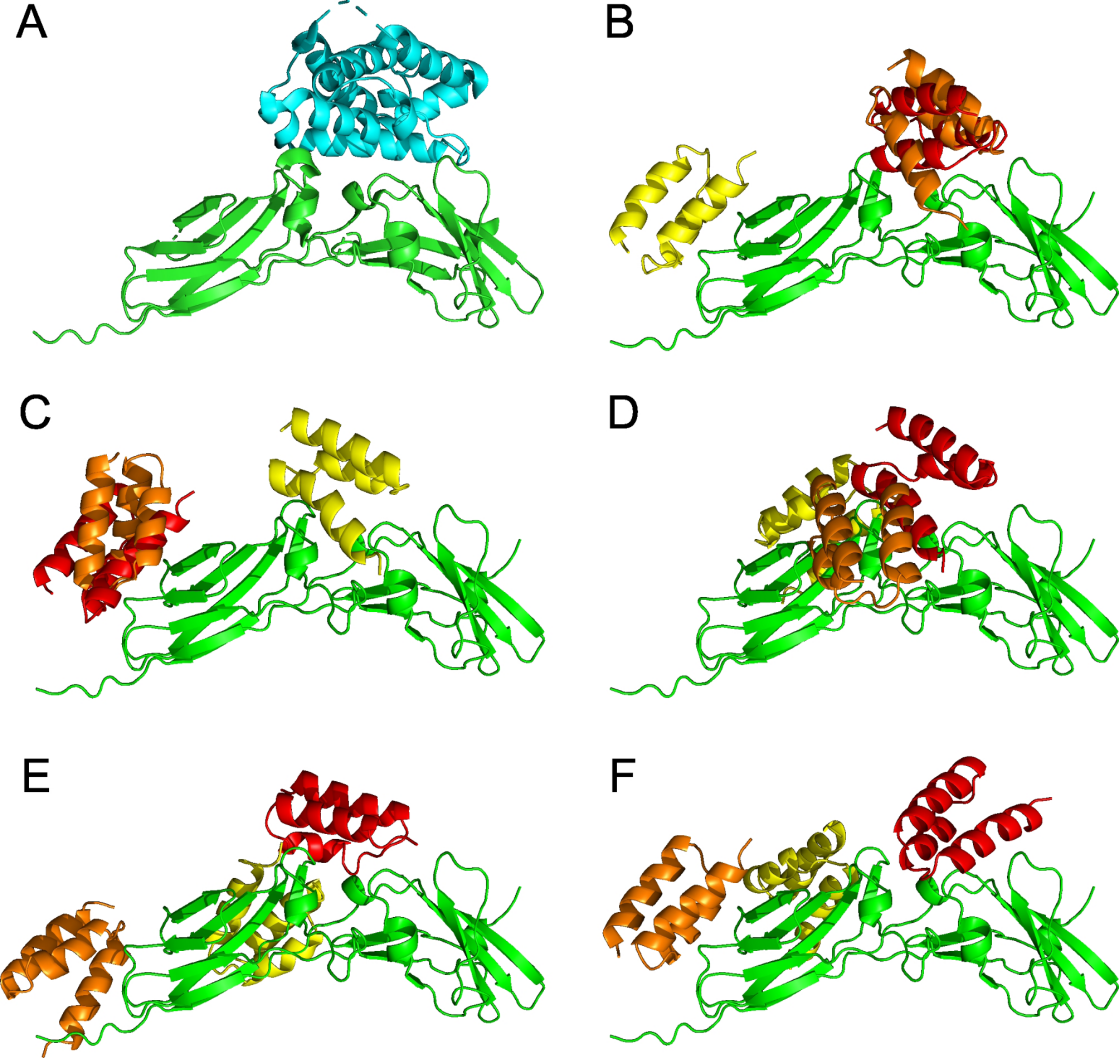
Prediction of most probable binding modes of ABR proteins to human IL-22R1. **(A)** The structure of the IL-22/IL-22R1 complex (pdb id 3dlq). The IL-22R1 is shown in green, the IL-22 in cyan. **(B-F)** Summary of ABR variants docking to the structure of the IL-22R1, the IL-22R1 is shown in green and the ABR variants in decreasing predicted order of binding as red, orange, and yellow cartoon; **(B)** shows results for ABR167, **(C)** for ABR089S, **(D)** for ABR089, **(E)** for ABR099S, and **(F)** shows the ABR099/IL-22R1 complex (doi 10.5281/zenodo.10418004)

To understand the details of interaction between the human IL-22R1 and ABR proteins we prepared in silico models of IL-22R1/ABR complexes. Docking results are summarized in Fig. 6 showing the three most probable predicted binding modes of ABR proteins to human IL-22R1 in decreasing predicted order of binding in red, orange, and yellow colors. The structure of the IL-22/IL-22R1 complex (pdb id 3dlq) is shown for comparison (Fig. 6A). The docking results for ABR167 (Fig. 6B), ABR089 (Fig. 6D), and ABR099 (Fig. 6F) suggest that their most probable binding modes overlap with the position occupied by the IL-22 cytokine in the experimental structure of the IL-22/IL-22R1 complex. This observation is consistent with the efficient inhibitory action of ABR167 and ABR089 variants as well as a slightly less efficient ABR099 action that could be attributed to its smaller overlap with the IL-22 binding site.

The ABR089 and ABR099 variants contain a Cysteine residue in their sequences, and this can potentially complicate production and stability of these variants. We have thus prepared models with Serine substitutions. The docking results for the ABR089S (Fig. 6C) predict that the most probable binding site does not overlap with the IL-22 and this variant no longer competes with IL-22 binding also experimentally. On the other hand, the results for the ABR099S variant (Fig. 6E) suggest an improved overlap with the IL-22 binding site. The atomic coordinates of the models are available in a PyMOL session on zenodo (10.5281/zenodo.10418004).

### Analysis of ABR protein-mediated IL-22R1 blocking function in murine model of experimentally induced colitis

Based on tested signaling inhibitory effect of ABR variants (Fig. 4A), we selected ABR167, as one of most promising, for in vivo testing in murine model of colitis induced by 2.5% DSS provided in drinking water.

**Fig. 7:**
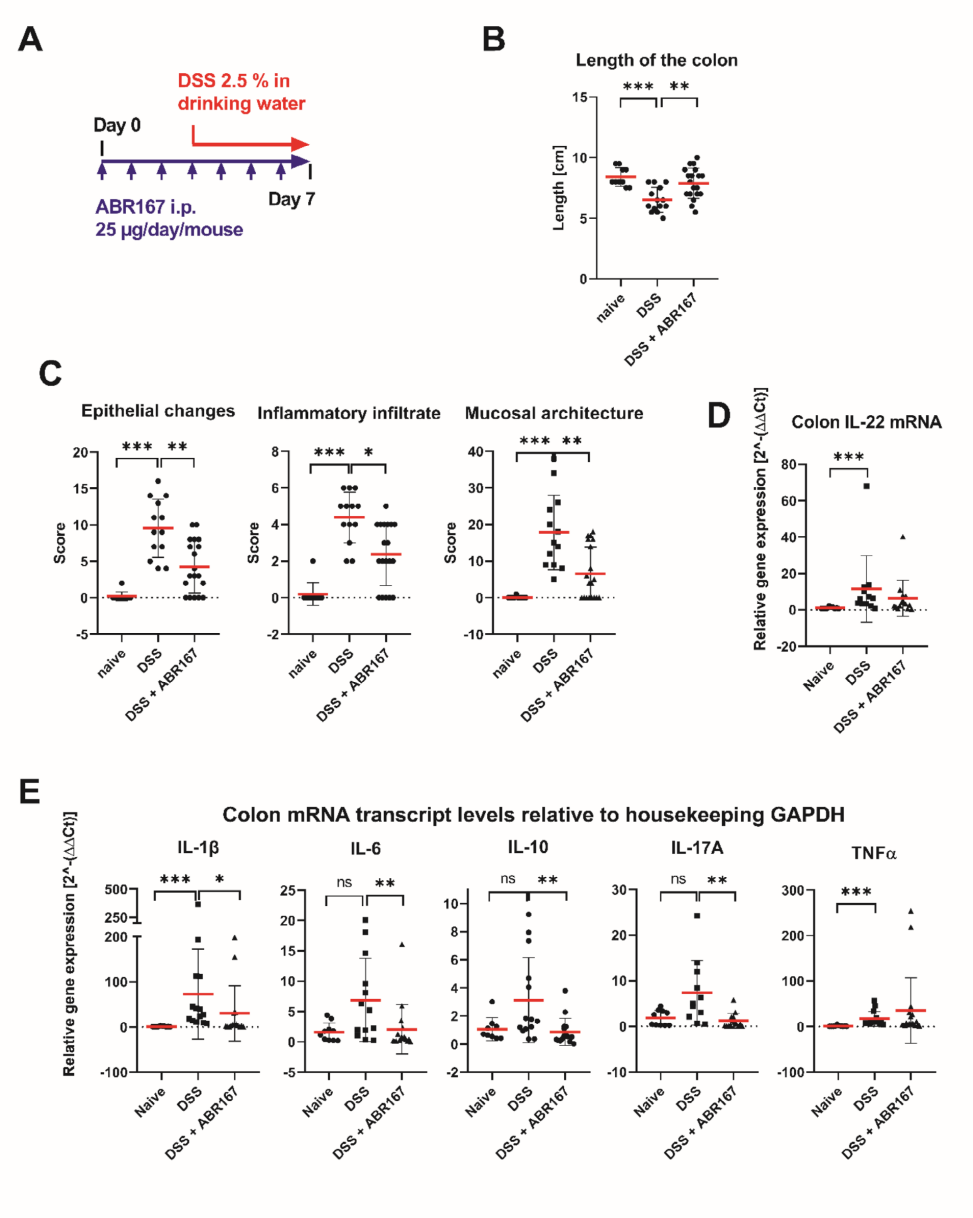
The effect of ABR167 administration on DSS-induced colitis severity. **(A)** Scheme of the DSS experiment. **(B)** Comparison of colon length in individually treated groups of mice. ABR167 protects colon against shortening caused by DSS exposure. **(C)** Histological assessment of colon tissues of naïve, DSS-exposed, and ABR167-treated DSS-exposed mice according to Erben et al. [47]. ABR167 significantly reduces inflammatory infiltrate, epithelial changes, and mucosal architecture according to classification specified in Materials and Methods. **(D)** Gene expression of IL-22 and **(E)** IL-1β, IL-6, IL-10, IL-17A, and TNFα determined by qRT-PCR. Statistical differences were analyzed by Kruskal-Wallis one-way ANOVA followed by Dunn´s multiple comparisons test. Means +/- SD are shown. *P < 0.05, **P < 0.01, ***P < 0.001

**Fig. 8:**
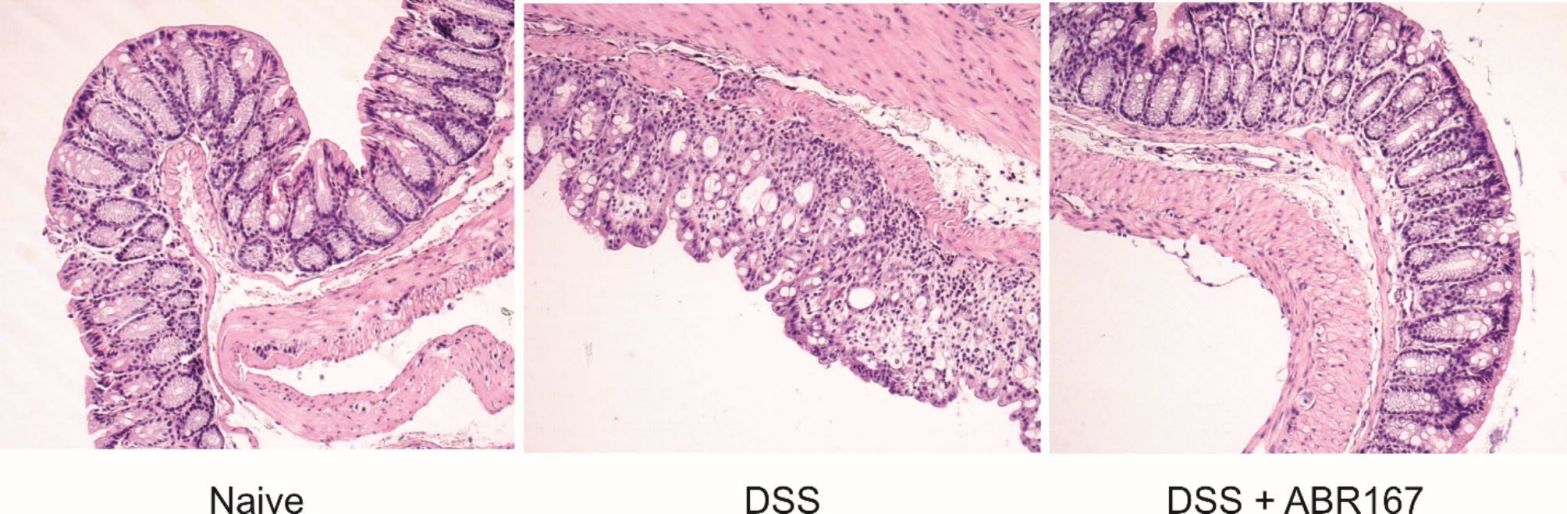
Representative examples of tissues dissected from colon. Naive – normal colon histology, normal structure of intestinal mucosa with preserved crypts, no active inflammation is present. DSS – erosion of intestinal mucosa with marked crypt loss, crypt hyperplasia and crypt irregularities, nonspecific granulation tissue formation with active inflammation. DSS + ABR167 – colon histology without marks of mucosal damage and without active inflammation

Time schedule of the experiment is shown on Fig. 7A and is summarized in methods. At time of experiment termination, the length of colon was measured. Compared to DSS-drinking (positive control) mice, administration of ABR167 significantly protected from shortening the length of the colon induced by DSS. In naïve mice the colon length was 8.4 ± 0.8 cm, in DSS drinking mice the length was 6.4 ± 0.9 cm and ABR167 treated mice exhibit mean length 7.9 ± 1.2 cm (Fig. 7B). The histology of terminal part of the colon was assessed on H&E-stained microscopic sections according to classification reported by Erben et al. [47]. The mean score for inflammatory infiltrate (assessed as leukocyte density in the lamina propria and deeper layers), epithelial changes (assessed as an increase in the number of epithelial cells in the longitudinal crypts relative to baseline and a decrease in Goblet cells, cryptitis) and mucosal architecture (assessed as epithelial defects, irregular presence of crypts, loss of crypts and blunting of villi) was calculated for each group (Fig. 7C). Detail classification of individual mice histology is presented in Table S5. Representative H&E pictures are shown in Fig. 8. ABR167-treated DSS-exposed mice exhibited significant reduction of gut histology alterations when compared to DSS-exposed control mice based on assessment of epithelial changes, inflammatory infiltrate, and mucosal architecture deterioration (Fig. 7C). Furthermore, the colon tissue samples were analyzed for the changes in the local expression of selected pro/anti-inflammatory markers (IL-1β, IL-6, IL-10, IL-17A, IL-22, and TNFα) by detecting their mRNA transcript levels using qRT-PCR. DSS-exposure induced significantly enhanced level of IL-22 transcript (Fig. 7D), which was not statistically reduced by ABR167 treatment although trend in IL-22 decrease in ABR167 treated mice correspond with less pronounced colon tissue deterioration (Fig. 7C). Among all other tested markers (Fig. 7E), IL-1β, IL-6, IL-10, IL-17A, and TNFα, mRNA transcript levels were increased although, due to inter-individual variability in tissue samples damage, the increase was achieving significance only for IL-1β and TNFα. The mRNAs expression in ABR167-treated DSS-exposed mice tend to shift toward normal values in comparison to DSS-exposed, otherwise untreated mice (Fig. 7E). Even here, the statistical significance was achieved for IL-1β, IL-6, IL-10, and IL-17A mRNA. We also tested systemic (serum) levels of cytokines corresponding to colon mRNA analyses (IL-1β, IL-6, IL-22, and TNFα) and other cytokines associated with systemic response (IL-4, IL-12p40, IL-18, and IL-23). After DSS-induced colitis we detected statistically significant increases in IL-22 and IL-18 concentration and insignificant but clearly visible trend in increase of IL-6 and IL-12p40 (Fig. S9).

## Discussion

Many cytokines exhibit pleiotropic effects, which could be positive or negative, depending on the biological environment [48, 49]. Thus, cytokines are strictly regulated through the host production of a cytokine-binding protein that docks or prevents interaction with cognate cellular receptors, permitting the site-specific regulation of their functions [50]. Although the pleiotropic nature of cytokines is essential for encouraging redundancy and feedback into immune responses, it is also a significant barrier to their use as therapeutic agents [51]. Although the combination of IL-22R1 and IL-10R2 subunits specifically mediates signaling by IL-22, both receptor subunits can be used by other cytokines such as IL-10, IL-26, IL-28 and IL-29, and IL-22R1 subunit also by IL-20 and IL-24 [52–54]. The receptor complex IL-22R1/IL-20R2, therefore, seems to mediate signaling events and effects that are very similar to those mediated by the IL-22R complex [13].

In presented experiments, binding of ABR variants to cell surface IL-22R1 was tested using HEK293T transiently transfected with human IL-22R1 by fluorescent microscopy and HEK-Blue IL22 reporter cell line stably transfected with IL-22R1 in flow cytometry experiments. HaCaT cells endogenously express IL-22R1 upon TNFα and IFNγ stimulation and were here used as induction-control. No IL-22R1 was detected for un-stimulated HaCaT cells, while stimulation with TNFα and IFNγ induced strong expression of IL-22R1 in 30–50% of cells (Fig. S6). Furthermore, HaCaT cells stimulated with TNFα and IFNγ were used to detect ABR variants binding to induced endogenously expressed IL-22R1. Seven ABR variants (ABR006, ABR023, ABR027, ABR089, ABR099, ABR102, and ABR167) exhibited binding to IL-22R1 transfectants (Fig. 3). Double staining carried out with ABR variants and anti-IL-22R1 antibody demonstrated that all ABR variants were able to recognize IL-22R1 on the surface of HaCaT cells with very similar binding patterns to anti-IL-22 antibody. This data indicates that ABR proteins specifically recognize cell surface expressed IL-22R1. Among all tested variants, ABR099 and ABR167 demonstrated the strongest binding and variant ABR167 was selected as a candidate for testing in murine model of ulcerative colitis.

Ulcerative colitis (UC) is a non-specific inflammatory bowel disease (IBD) resulting from a complex interplay between environmental, microbial, and genetic factors, leading to abnormal immunological response with intestinal inflammation. DSS is a water-soluble, negatively charged sulfated polysaccharide of which 40–50 kDa fraction is able to induce colitis in C57BL/6 mice, closely resembling human UC [55]. DSS-colitis represents an acute murine model of colitis in which cytokines of IL-17, IL-23, and IL-22 group are involved [56–58]. IL-17F deficiency resulted in reduced pathology of DSS-induced colitis in mice, whereas IL-17A deficiency was associated with more severe disease [57]. Both cytokines have a redundant but unequal pathogenic role in gut inflammation and balanced inhibition of IL-17A and IL-17F represents promising goal for therapy development in chronic colitis [59]. The IL-23 has emerged as another crucial cytokine and promising therapeutic target in IBD. IL-23 is a heterodimeric cytokine comprised of IL-23p19 and IL-12p40 [60]. In DSS-induced colitis IL-23R signaling by mature lymphocytes reduced pathology but in the absence of lymphocytes IL-23 signaling promotes pathology [56], indicating dichotomy in the function of the IL-23 pathway in adaptive versus innate immune compartments [56, 61]. In innate (lymphocyte independent) colitis model, IL-23R-related pathology was associated with IL-22 signaling, as neutralization of IL-22 exhibited protectivity [61]. The depletion of innate lymphoid cells nILC2, ILC1, and ILC3 eliminated the majority of IL-22 production in the colon lamina propria of DSS-treated Rag2^−/−^ mice indicating that nILC2, ILC1, and ILC3 are the major IL-23 responsive innate cells in this model [62, 63], although others have also reported IL-23-dependent IL-22 production by neutrophils [64, 65]. In the present project, we tested the effect of selected IL-22R1 blocker ABR167 as a representative of the most promising candidates, based on their antagonistic activity on IL-22R1-mediated signaling tested on HEK-Blue IL22 reporter cells (Fig. 4A). Here we used oral DSS administration-induced colitis in C57BL/6 mice and showed that intraperitoneal administration of ABR167 (Fig. 7A) could significantly reduce subsequent colitis development as detected by colon length and colon histology (Fig. 7B, C). In tissue samples of colon from DSS-exposed control mice, an increased IL-22 mRNA transcript was confirmed (Fig. 7D) which justifies the usage of ABR167 for IL-22R1 targeting, although we are aware of limitations due to the pleithropy and promiscuity of cytokine to cytokine receptor interaction [13, 52–54]. In treated mice, the increase in IL-22 production was less pronounced which, in accordance with only modest signs of histology inflammation in ABR167-treated mice, indicates a weaker activation of IL-22 producing cells in an affected colon. In accordance, colon production of other pro/anti-inflammatory markers, such as IL-1β, IL-6, and IL-17A or IL-10, was reduced in ABR167-treated mice compared to control DSS-exposed mice (Fig. 7E). Although significantly, TNFα transcript increase was very modest after DSS and the effect of ABR167 treatment on TNFα expression was not significant, which could be linked to used DSS model properties with only moderate level of induced colitis due to experimental protocol setup (only 4 days of 2.5% DSS exposure), which we opted in present study to detect even moderate improvement in colitis after ABR167 treatment. As we expected, an increase in the systemic (serum) levels of pro-inflammatory cytokines (IL-6, IL-22, IL-12p40, and IL-18) was, therefore, only moderate (Fig. S9). Based on these results, it seems to be reasonable to design further colitis-inducing experiments with an extended duration in combination with other triggering agents such as 2, 4, 6-trinitrobenzenesulfonic acid (TNBS). ABR blockers-based strategy can be used as a proof-of-concept for future colitis-modifying drug development. For example, recently published NEF protein blockers of human IL-6 receptor, derived from the ABD scaffold, exhibited a substantial inflammatory reduction in DSS-induced murine colitis model [66].

While ABR167 protein is free of Cysteine residues, ABR089 and ABR099 variants contain Cysteine in the randomized position. This leads to the formation of dimers that could, in particular cases, sterically restrict the accessibility of ABR to the binding site on IL-22R1 and, thus, affect the affinity and blocking potential. Therefore, we used in silico modeling to predict, and experimentally verify, function of the Cysteine-substituted ABR variants. Our in silico modeling and docking studies provide valuable insights into the binding interactions between human IL-22R1 and ABR proteins. These findings not only shed light on the structural aspects of this interaction but also highlight the potential significance of amino acid substitutions, such as Serine substitutions, in modulating the binding behavior of ABR variants. Analysis of the crystal structure of the IL-22/IL-22R1 complex (PDB ID 3dlq, Fig. 6A) revealed a tight interaction between W208 residue of the IL-22R1 and M172 of the IL-22. A nearly identical interaction is reproduced in the model of the ABR099S variant where the M37 residue interacts with the W208 of the IL-22R1. This interaction was not formed before the C/S substitution and the ABR099S thus successfully combines favorable interactions with removing the unwanted tendency for oligomerization of the initial Cysteine variant ABR099. On the other hand, the Cysteine residue in the ABR089 plays a similar role as the Methionine and is involved in a direct interaction with the W208 of the receptor. This can explain the loss of activity after the C/S substitution and although the ABR089 variant has a potential for oligomerization the initial sequence should be used. Further experimental investigations are warranted to validate and extend these computational findings, ultimately advancing our understanding of the biological implications of these interactions. In silico modeling thus opens window for the identification of sequentially improved candidates for future in vivo testing.

## Conclusion

In summary, we report here on the generation of unique small binding proteins that might function as human IL-22R1 antagonists with therapeutic potential. These can be useful as a molecular clue for designing novel IL-22R1-targeted therapies. Our results further ascertain the importance of IL-22 signaling axis in the gut inflammation. As ABD-derived blockers can be effectively produced in genetically engineered *L. lactis* probiotic strains, it offers a promising avenue for orally administrated IL-22R1-targeted therapy for gut inflammatory conditions such as ulcerative colitis.

## Electronic supplementary material

Below is the link to the electronic supplementary material.

Supplementary Material 1

## Data Availability

No datasets were generated or analysed during the current study.

## Abbreviations

ABD: Albumin-binding domain
BSA: Bovine Serum Albumin
DEPC: Diethyl pyrocarbonate
DMEM: Dulbecco’s modified Eagle’s medium
DSS: Dextran sulfate sodium
E. coli: Escherichia coli
EDTA: Ethylenediaminetetraacetic acid
ELISA: Enzyme-linked immunosorbent assay
FBS: Fetal bovine serum
FCS: Fetal Calf Serum
FDA: Food and Drug Administration
FDA: Food and Drug Administration
FITC: Fluorescein isothiocyanate
GVHD: Graft versus host disease
HBSS: Hank’s Balanced Salt Solution
HEPES: 4-(2-Hydroxyethyl)piperazine-1-ethanesulfonic acid
HRP: Horseradish peroxidase
IBD: Inflammatory bowel disease
IFN-γ: Interferon-gamma
IL-10: Interleukin-10
IL-22: Interleukin-22
IL-22R1: Interleukin-22 receptor alpha
IL-6: Interleukin-6
ILC3: Innate lymphoid cell type 3
IPTG: isopropyl β-d-1-thiogalactopyranoside
IPTG: Isopropyl β-d-1-thiogalactopyranoside
LPS: Lipopolysaccharide
mAb: Monoclonal antibody
MFI: Mean fluorescence intensities
NIR: Near-infrared
pAb: polyclonal antibody
RBS: Ribosome binding site
SEAP: Secreted embryonic alkaline phosphatase
STAT: Signal transducer and activator of transcription
T7p: Bacteriophage T7 RNA Polymerase Promoter
TMB: 3,3’,5,5’-Tetramethylbenzidine
TNF-α: Tumor necrosis factor-alpha
