# Supplementary material for: Human IL-22 receptor-targeted small protein antagonist suppress murine DSS-induced colitis
Source: Cell Commun Signal. 2024 Oct 1;22:469. doi: 10.1186/s12964-024-01846-w (PMC11446014; doi:10.1186/s12964-024-01846-w)
Supplement: Supplementary file 1 — Supplementary Material 1 [file 12964_2024_1846_MOESM1_ESM.pdf]

Supplementary information

Human IL-22 receptor-targeted small protein antagonist suppress murine DSS-induced colitis

Milan Kuchař[1#], Kristýna Sloupenská[2#], Leona Rašková Kafková[2*], Yaroslava Groza[1], Jozef Škarda[3], Petr Kosztyu[2], Marie Hlavničková[1], Joanna M. Mierzwicka[1], Radim Osička[4], Hana Petroková[1], Stephen I. Walimbwa[2], Shiv Bharadwaj[1], Jiří Černý[5], Milan Raška[2], Petr Malý[1*].

[1]Laboratory of Ligand Engineering, Institute of Biotechnology of the Czech Academy of Sciences, BIOCEV Research Center, Prumyslova 595, 252 50 Vestec, Czech Republic

[2]Department of Immunology, Faculty of Medicine and Dentistry, Palacky University Olomouc, Hnevotinska 3, 779 00, Olomouc, Czech Republic

[3]Department of Pathology, University Hospital Ostrava and Faculty of Medicine, University of Ostrava, Ostrava, Czech Republic

[4]Laboratory of Molecular Biology of the Bacterial Pathogens, Institute of Microbiology of the Czech Academy of Sciences, Vídeňská 1083, 14220 Prague, Czech Republic

[5]Laboratory of Structural Bioinformatics of Proteins, Institute of Biotechnology of the Czech Academy of Sciences, BIOCEV Research Center, Prumyslova 595, 252 50 Vestec, Czech Republic

[#] These authors contributed equally to this work

[*]Authors for correspondence

Petr Malý, Institute of Biotechnology of the Czech Academy of Sciences, Prumyslova 595, 252 50 Vestec, Czech Republic; Phone: +420 325873763, E-mail: petr.maly@ibt.cas.cz

Leona Rašková Kafková, Faculty of Medicine and Dentistry, Palacky University Olomouc, Hnevotinska 3, 779 00, Olomouc, Czech Republic; E-mail: leona.raskova@upol.cz

S1. Methodology

Table S1. Forward and reverse sequence for the ABR domain used in the assembly of ABR library.

**Forward ABRLIB-setB1c:**	TAGCTGAAGCTAAAGTCTTAGCTAACAGAGAACTTGACAAATATGGAGTAAGTGAC
**Reverse ABRLIB-setB2c**:	ACCGCGGATCCAGGTAAMNNAGCTAAAATMNNATCTATMNNMNNTTTTACMNNMNNAACMNNMNNGGCMNNGTTGATMNNGTTCTTGTAMNNGTCACTTACTCCATATTTGTC

Table S2. List of primers used in murine DSS induced colitis.

**Gene**	Primer	Sequence (5´ to 3´)
**GAPDH**	Forward	CGTCCCGTAGACAAAATGGT
	Reverse	TCAATGAAGGGGTCGTTGAT
**IL-1β**	Forward	TGCCACCTTTTGACAGTGATG
	Reverse	ATGTGCTGCTGCGAGATTTG
**TNFα**	Forward	AAGCCTGTAGCCCACGTCGTA
	Reverse	AGGTACAACCCATCGGCTGG
**IL-6**	Forward	ACAAAGCCAGAGTCCTTCAGAG
	Reverse	GCCACTCCTTCTGTGACTCC
**IL-10**	Forward	TGAATTCCCTGGGTGAGAAG
	Reverse	TGGCCTTGTAGACACCTTGG
**IL-22**	Forward	GGTGACGACCAGAACATCCA
	Reverse	GACGTTAGCTTCTCACTTTCCTT
**IL-17A**	Forward	TTTTCAGCAAGGAATGTGGA
	Reverse	TTCATTGTGGAGGGCAGAC

S2. Results

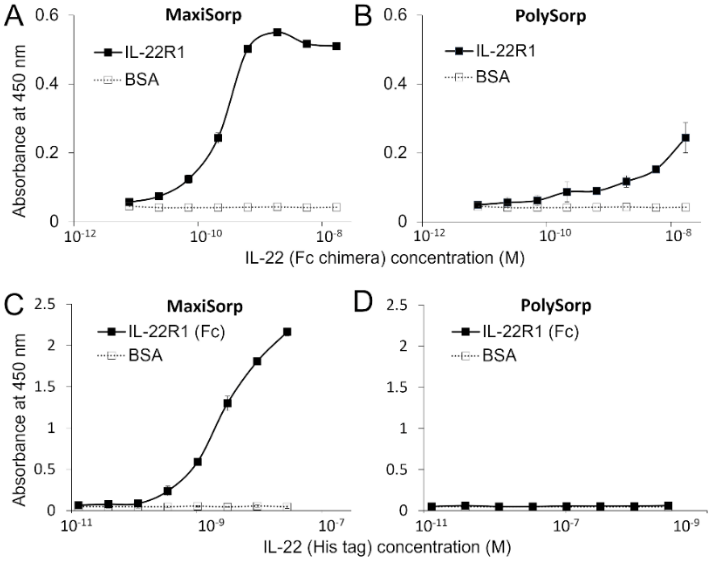

Fig. S1. Binding of IL-22 cytokine to human IL-22R1 tested by ELISA. IL-22-IgG Fc cytokine binds to IL-22R1 on MaxiSorp immuno-plate (A) or Polysorp plate (B). Binding of His-IL-22 to human IL-22R1-IgG Fc was tested by MaxiSorp (C) and PolySorp (D). For detection, mouse IgG1 Anti-Hu IgG (Fc)-HRP was used (A, B) or anti-His monoclonal antibody-HRP (C, D).

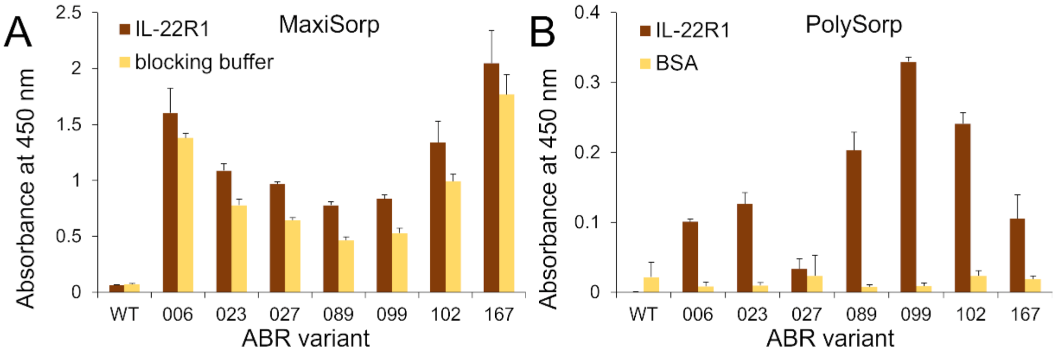

Fig. S2. Selection of IL-22R1-binding ABR variants using ELISA. Positive variants and ABDwt as a negative control are shown in (A) and (B). For screening, lysates containing in vivo biotinylated protein variant diluted in PBS (15 000 times, for protein concentration to be ~1nM) were tested for binding to immobilized IL-22R1 (2.5 μg/ml) in comparison to only blocked wells, on MaxiSorp plate with blocking by PierceTM Protein-Free Blocking Buffer (A) or on PolySorp plate with blocking by BSA (B). Binding was detected by streptavidin-HRP (1: 10 000) and results represent average values with standard deviation error bars.

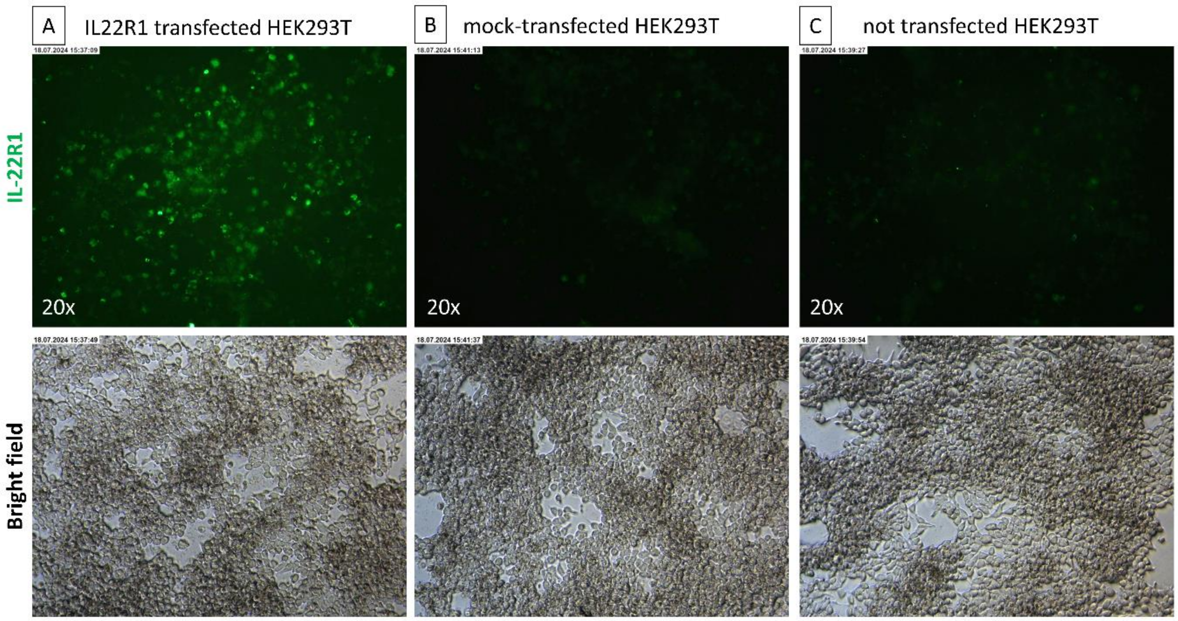

Fig. S3. Verification of the specificity of anti-IL-22R1 antibody on HEK293T cells transfected with IL22R1-pcDNA6 vector (A) in comparison to the staining performed on cells transfected with empty pcDNA6 plasmid (B) and non-transfected cells (C). Immunofluorescence staining was performed using primary polyclonal rabbit anti-IL22R1 antibody and secondary mouse anti-rabbit Alexa Fluor 488 conjugate. Magnification is 20x.

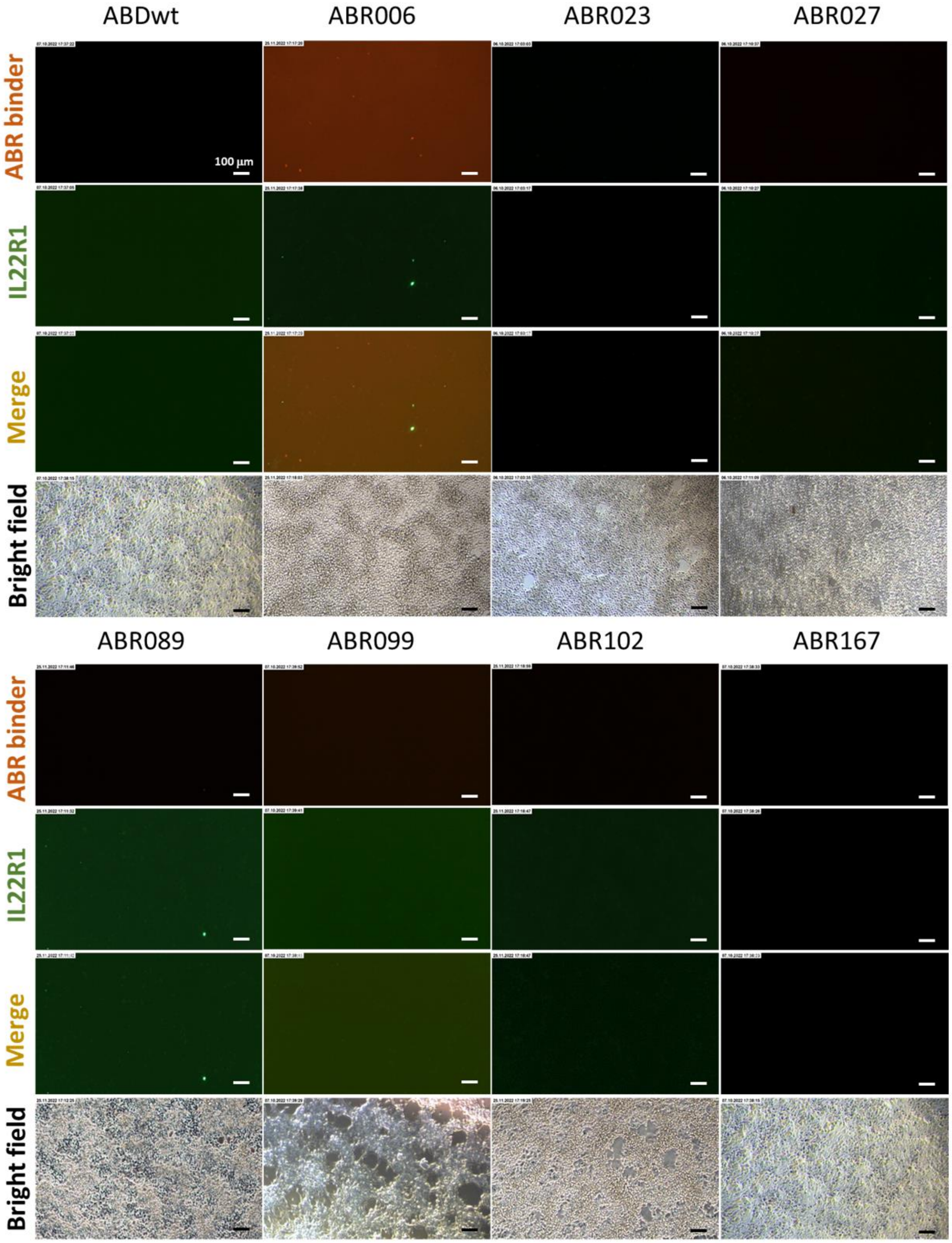

Fig. S4. Control negative staining of ABR proteins to mock-transfected HEK293T cells. The loss of ABR proteins´ binding correlates with the loss of anti-IL-22R1 antibody staining. Detection of biotinylated ABR variants was done using Streptavidin AlexaFluor 568 conjugate. IL22R1 expression was detected by rabbit polyclonal anti-IL22R1 antibody and mouse anti-rabbit AlexaFluor 488 antibody conjugate used as a secondary reagent. Magnification is 10x.

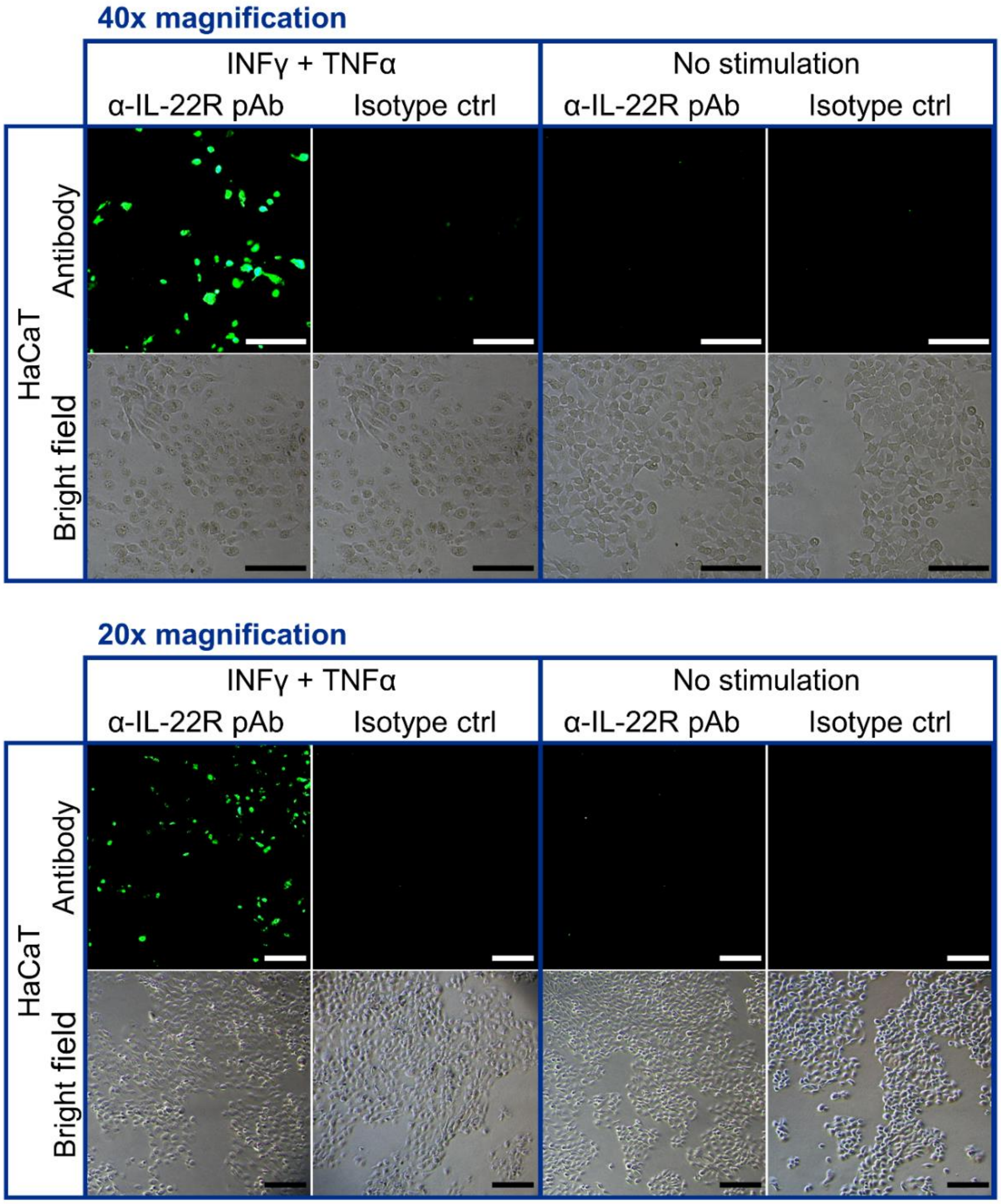

Fig. S5. Detection of IL-22R1 on HaCaT cells stimulated with TNFα and IFNγ. HaCaT cells were stimulated with TNFα and IFNγ for 24 h. TNFα- and IFNγ-induced IL-22R1 expression was detected using rabbit anti-IL-22 pAb and secondary AF 488-conjugated antibody. Non-stimulated HaCaT cells do not express detectable amount of IL-22R1. Rabbit polyclonal isotype control was used with stimulated and non-stimulated HaCaT cells to demonstrate anti-IL-22 pAb specificity.

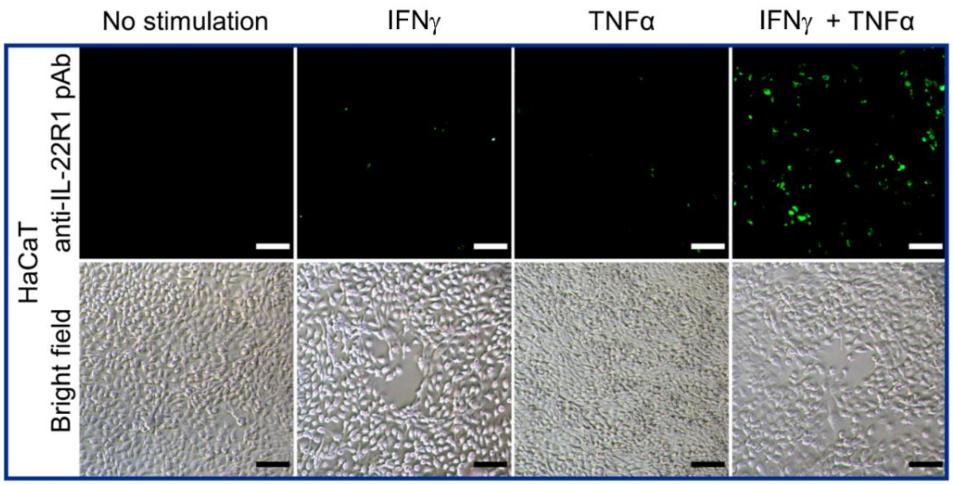

Fig. S6. Detection of IL-22R1 on HaCaT cells stimulated with TNFα and IFNγ. HaCaT cells were stimulated with TNFα, IFNγ, and a combination of both for 24 h. TNFα- and IFNγ-induced IL-22R1 expression was detected using rabbit anti-IL-22 pAb-AF 488 (green). Non-stimulated HaCaT cells do not express detectable amount of IL-22R1. TNFα alone and IFNγ alone stimulate barely detectable IL-22R1 expression. Combination of TNFα and IFNγ stimulated expression of IL-22R1 detectable in 30-50% of cells.

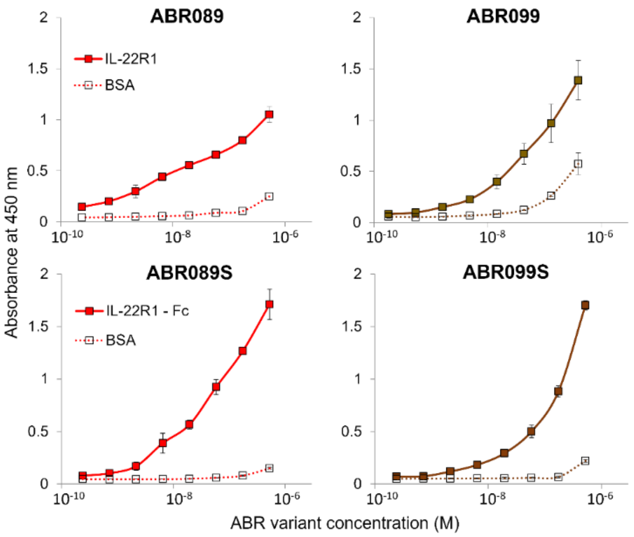

Fig. S7. Binding of ABR089 and ABR099 proteins containing cysteine in the randomized position and their modified versions with replacement of cysteine for serine (ABR089S and ABR099S) in ELISA. PolySorp plate was coated with IL-22R1 or IL-22R1-Fc chimera (2 mg/mL) and blocked with 1% BSA. Binding of ABR variants was detected by streptavidin-HRP (1: 10 000) and resulted curves represent average values with standard deviation error bars.

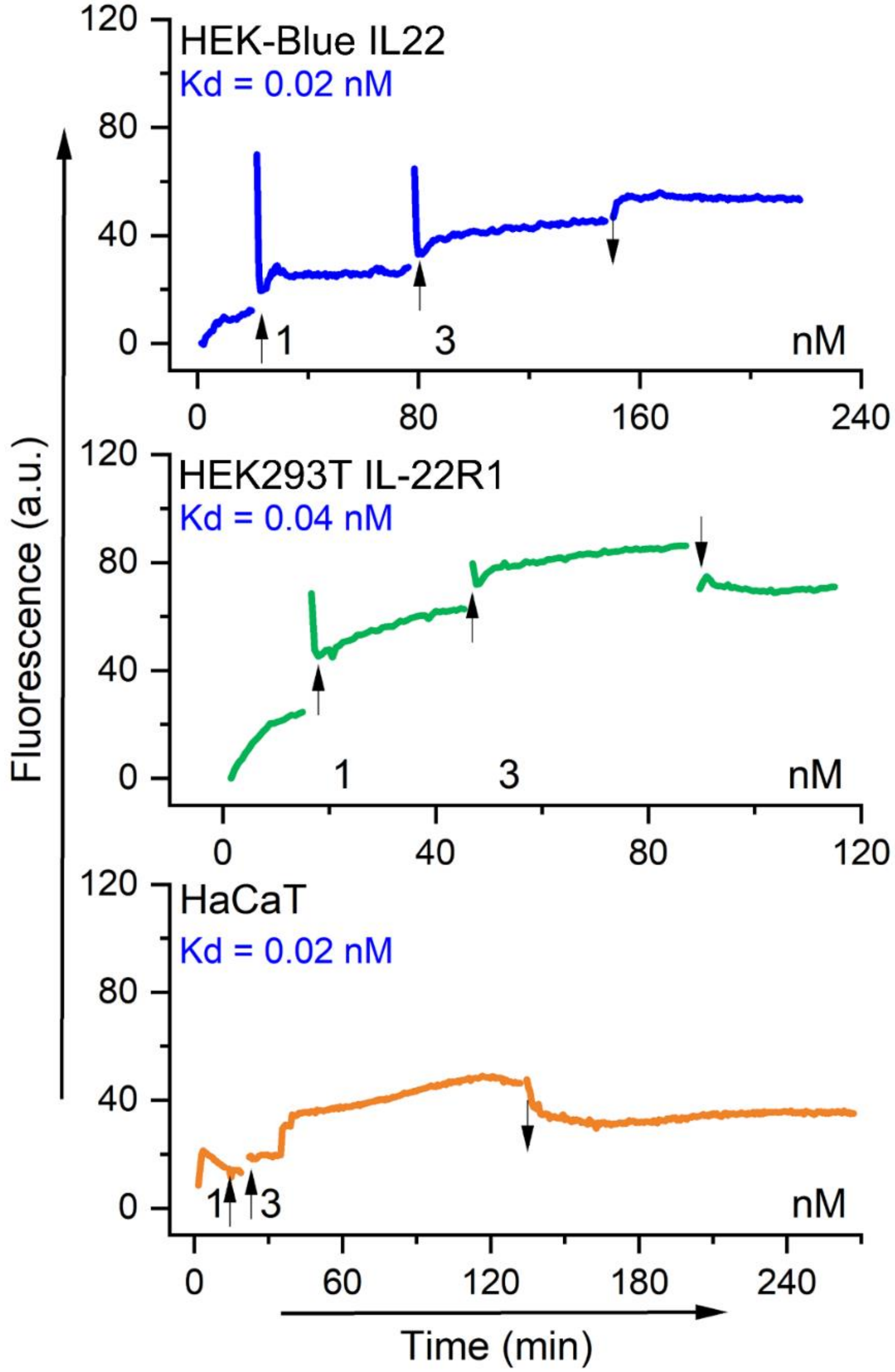

Fig S8. Binding of anti-IL-22R1 antibody to HEK-Blue IL22 cells, HEK293T cells transfected with IL-22R1-pcDNA6 and HaCaT cells using LigandTracer Green. Binding curves are presented for all three cell types utilized in experiments. Shown results are after subtraction of the background from the signal obtained for the cells.

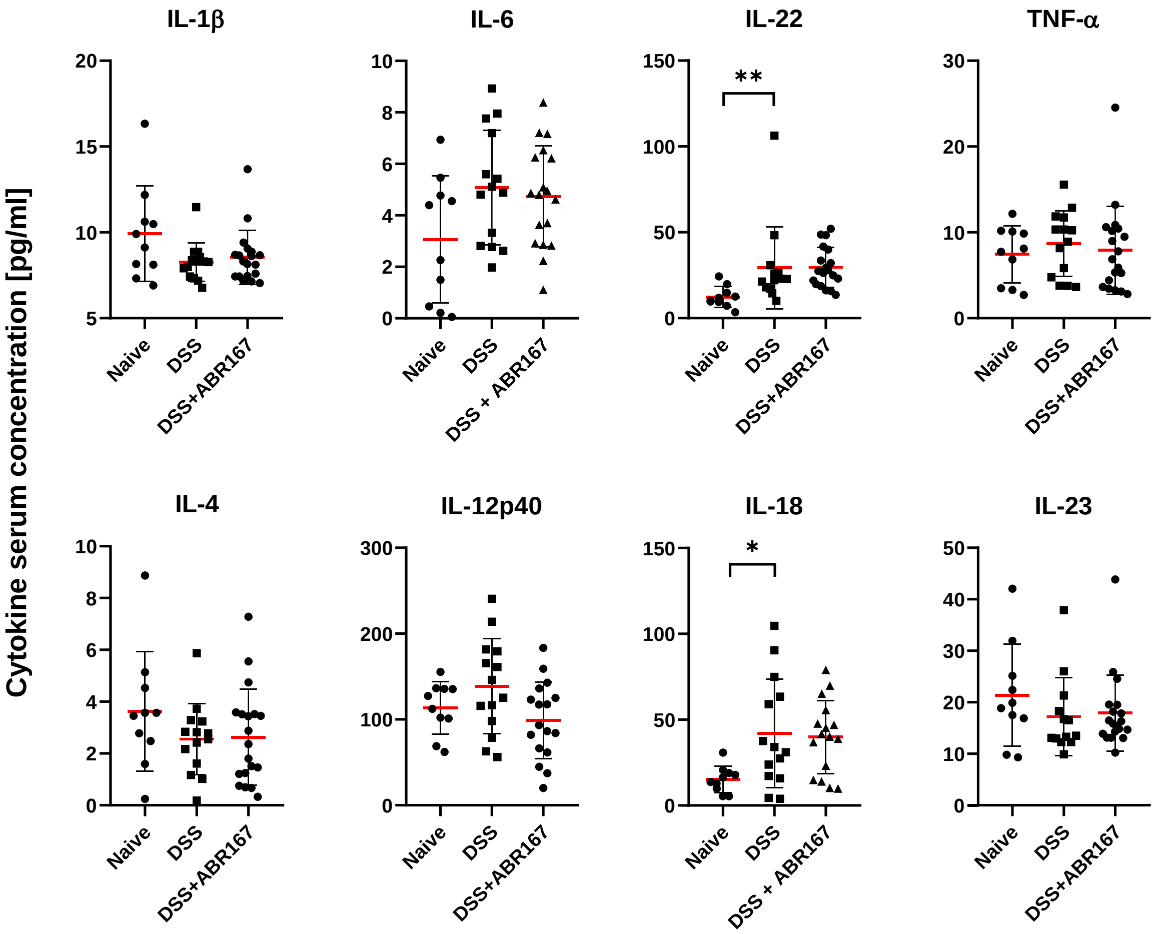

Fig. S9. The levels of serum cytokines in naive, DSS treated and DSS + ABR167-treated mice.

Table S3. Binding affinity was measured for three types of cells used in experiments. Measurements were performed with Ligand Tracer Green and anti-IL-22R1-APC conjugated antibody was used for detection of IL-22R1 on the cells.

**Anti-IL-22R1-APC conjugated antibody**			
**Cell line**	**KD (M)**	**ka (1/M*s))**	**kd (1/s)**
HEK-Blue IL22	2.14×10^{-11}	2.95×10^{4}	6.33×10^{-7}
HEK293T-IL-22R1-pcDNA6	4.26×10^{-11}	7.45×10^{4}	3.17×10^{-6}
HaCaT/IFNγ + TNFα activated	2.09×10^{-11}	1.66×10^{5}	3.46×10^{-6}

Table S4. Comparative table with kinetic parameters calculated for ABR variants. Kinetic parameters (KD, ka and kd) were evaluated using IL-22R1 transfected HEK293T cells (upper table) and HaCaT cells stimulated with TNFα and IFNγ 10 ng/ml (lower table). Kinetics for 9 ABR variants was measured on transfected HEK293T cells and for 5 selected ABR variants also on activated HaCaT cells.

**HEK293T cells transfected with IL-22R1-pcDNA6**			
**ABR variants**	**KD (M)**	**ka (1/M*s))**	**kd (1/s)**
**ABR006**	4.26×10^{-8}	6.07×10^{3}	2.58×10^{-4}
**ABR023**	6.87×10^{-9}	4.47×10^{4}	3.07×10^{-5}
**ABR027**	3.44×10^{-8}	9.41×10^{4}	3.24×10^{-3}
**ABR089**	3.37×10^{-9}	5.62×10^{5}	1.90×10^{-3}
**ABR089S**	1.09×10^{-8}	1.43×10^{4}	1.56×10^{-4}
**ABR099**	1.06×10^{-9}	8.18×10^{4}	8.64×10^{-5}
**ABR099S**	6.71×10^{-9}	2.44×10^{4}	1.64×10^{-4}
**ABR102**	9.38×10^{-9}	4.03×10^{4}	3.78×10^{-4}
**ABR167**	7.32×10^{-9}	6.66×10^{4}	4.88×10^{-4}
**HaCaT cells**			
**ABR006**	N/A	N/A	N/A
**ABR023**	5.87×10^{-8}	1.01×10^{4}	5.94×10^{-4}
**ABR027**	N/A	N/A	N/A
**ABR089**	9.73×10^{-9}	2.63×10^{5}	2.55×10^{-3}
**ABR089S**	N/A	N/A	N/A
**ABR099**	3.04×10^{-9}	6.20×10^{4}	1.88×10^{-4}
**ABR099S**	N/A	N/A	N/A
**ABR102**	2.59×10^{-8}	1.65×10^{5}	4.26×10^{-3}
**ABR167**	5.47×10^{-9}	1.75×10^{4}	9.54×10^{-5}

Table S5. Overview of histology assessments for individual experimental mice.

Group	Mouse number	SCORE Inflammation-severity	SCORE Inflammation-extent	Inflam. Infiltr.	SCORE Hyperplasia	SCORE Goblet cell loss	SCORE Cryptitis	SCORE Crypt abscesses	SCORE Erosion	Epithelial changes	SCORE Ulceration	SCORE Granulation tissue	SCORE Irregular crypts	SCORE Crypt loss	SCORE Vilous blunting	Extent of damage	Mucosal architecture
		minimal 1, mild 2, moderate 3, marked 4	mucosal 1, submucosal 2, transmural 3	SCORE	minimal 1, mild 2-3, moderate 3-4, marked 4-5	minimal 1, mild 2-3, moderate 3-4, marked 4	2/3	3-5	1-4	SCORE	4/5	4/5	4/5	4/5	(mild 1, moderate 2-4, marked 5)	1 focal, 2 focal marked	SCORE
Naive	882	x	x	0	x	x	x	x	x	0	x	x	x	x	x	x	0
Naive	883	x	x	0	x	x	x	x	x	0	x	x	x	x	x	x	0
Naive	884	x	x	0	x	x	x	x	x	0	x	x	x	x	x	x	0
Naive	885	x	x	0	x	x	x	x	x	0	x	x	x	x	x	x	0
Naive	886	x	x	0	x	x	x	x	x	0	x	x	x	x	x	x	0
Naive	669	x	x	0	x	x	x	x	x	0	x	x	x	x	x	x	0
Naive	670	x	x	0	x	x	x	x	x	0	x	x	x	x	x	x	0
Naive	671	x	x	0	x	x	x	x	x	0	x	x	x	x	x	x	0
Naive	672	x	x	0	x	x	x	x	x	0	x	x	x	x	x	x	0
Naive	673	1	1	2	1	1	x	x	x	2	x	x	x	x	1	1	1
DSS	887	Damaged sample/not analyzed															
DSS	888	2	2	4	2	3	x	x	x	5	x	4	4	x	x	1	8
DSS	889	3	2	5	2	5	x	x	2	9	x	4	4	5	1	1	14
DSS	890	3	2	5	3	4	2	3	2	14	x	x	4	4	1	1	9
DSS	891	3	2	5	3	4	2	3	2	14	x	x	4	4	1	1	9
DSS	892	2	2	4	3	2	2	x	x	7	x	x	4	x	1	1	5
DSS	893	3	3	6	3	3	2	x	1	9	x	5	5	5	2	2	34
DSS	894	3	2	5	3	4	x	x	4	11	x	5	4	5	4	1	18
DSS	895	3	3	6	3	4	2	x	4	13	x	5	4	5	1	1	15
DSS	674	2	1	3	1	3	x	x	x	4	x	x	4	4	2	2	20
DSS	676	1	1	2	2	3	2	x	x	7	x	x	4	5	3	1	12
DSS	675	1	1	2	1	1	2	x	x	4	x	x	4	4	1	2	18
DSS	678	4	2	6	5	4	3	x	4	16	x	4	5	5	5	2	38
DSS	677	3	1	4	4	4	3	x	x	11	x	x	5	4	4	2	26
ABR167	932	x	x	0	x	x	x	x	x	0	x	x	x	x	x	x	0
ABR167	933	2	2	4	3	1	2	x	x	6	x	x	4	x	x	1	4
ABR167	934	2	2	4	3	3	2	x	x	8	x	x	4	x	1	1	5
ABR167	935	x	x	0	x	x	x	x	x	0	x	x	x	x	x	x	0
ABR167	936	x	x	0	x	x	x	x	x	0	x	x	x	x	x	x	0
ABR167	937	x	x	0	x	x	x	x	x	0	x	x	x	x	x	x	0
ABR167	938	x	x	0	x	x	x	x	x	0	x	x	x	x	x	x	0
ABR167	939	2	2	4	3	3	2	x	2	10	x	5	4	5	2	1	16
ABR167	940	3	2	5	3	3	2	x	x	8	x	4	4	4	2	1	14
ABR167	699	1	1	2	2	2	x	x	x	4	x	4	4	x	x	1	8
ABR167	700	1	1	2	2	1	x	x	x	3	x	x	x	x	x	1	0
ABR167	701	1	1	2	1	1	x	x	x	2	x	x	4	x	2	1	6
ABR167	749	1	1	2	2	1	x	x	x	3	x	x	x	x	x	1	0
ABR167	703	3	1	4	3	3	2	x	2	10	x	4	4	4	4	1	16
ABR167	704	2	1	3	2	1	x	x	x	3	x	x	x	x	x	1	0
ABR167	705	3	1	4	2	1	2	x	2	7	x	4	5	4	4	1	17
ABR167	706	2	1	3	2	2	2	x	1	7	x	4	4	4	5	1	17
ABR167	707	3	1	4	2	2	2	x	2	8	x	5	5	4	4	1	18
ABR167	**708**	**1**	**1**	**2**	**1**	**1**	**x**	**x**	**x**	**2**	**x**	**x**	**4**	**x**	**x**	**1**	**4**
